# A novel DSP zebrafish model reveals training- and drug-induced modulation of arrhythmogenic cardiomyopathy phenotypes

**DOI:** 10.1038/s41420-023-01741-2

**Published:** 2023-12-06

**Authors:** Rudy Celeghin, Giovanni Risato, Giorgia Beffagna, Marco Cason, Maria Bueno Marinas, Mila Della Barbera, Nicola Facchinello, Alice Giuliodori, Raquel Brañas Casas, Micol Caichiolo, Andrea Vettori, Enrico Grisan, Stefania Rizzo, Luisa Dalla Valle, Francesco Argenton, Gaetano Thiene, Natascia Tiso, Kalliopi Pilichou, Cristina Basso

**Affiliations:** 1 Department of Cardiac-Thoracic-Vascular Sciences and Public Health, University of Padova, Padova, 35128 Italy; 2 Department of Biology, University of Padova, Padova, 35131 Italy; 3 Neuroscience Institute, Italian National Research Council (CNR), Padova, 35131 Italy; 4 Department of Biotechnology, University of Verona, Verona, 37134 Italy; 5 School of Engineering, London South Bank University, London, SE1 0AA UK

**Keywords:** Disease genetics, Ventricular tachycardia

## Abstract

Arrhythmogenic cardiomyopathy (AC) is an inherited disorder characterized by progressive loss of the ventricular myocardium causing life-threatening ventricular arrhythmias, syncope and sudden cardiac death in young and athletes. About 40% of AC cases carry one or more mutations in genes encoding for desmosomal proteins, including Desmoplakin (Dsp). We present here the first stable Dsp knock-out (KO) zebrafish line able to model cardiac alterations and cell signalling dysregulation, characteristic of the AC disease, on which environmental factors and candidate drugs can be tested. Our stable Dsp knock-out (KO) zebrafish line was characterized by cardiac alterations, oedema and bradycardia at larval stages. Histological analysis of mutated adult hearts showed reduced contractile structures and abnormal shape of the ventricle, with thinning of the myocardial layer, vessels dilation and presence of adipocytes within the myocardium. Moreover, TEM analysis revealed “pale”, disorganized and delocalized desmosomes. Intensive physical training protocol caused a global worsening of the cardiac phenotype, accelerating the progression of the disease. Of note, we detected a decrease of Wnt/β-catenin signalling, recently associated with AC pathogenesis, as well as Hippo/YAP-TAZ and TGF-β pathway dysregulation. Pharmacological treatment of mutated larvae with SB216763, a Wnt/β-catenin agonist, rescued pathway expression and cardiac abnormalities, stabilizing the heart rhythm. Overall, our Dsp KO zebrafish line recapitulates many AC features observed in human patients, pointing at zebrafish as a suitable system for in vivo analysis of environmental modulators, such as the physical exercise, and the screening of pathway-targeted drugs, especially related to the Wnt/β-catenin signalling cascade.

## Introduction

Arrhythmogenic Cardiomyopathy (AC) is a hereditary cardiac disease characterized by progressive replacement of cardiomyocytes by fibro-fatty tissue, starting from the epicardium and extending toward the endocardium [1, 2]. Because of this transmural damage, thinning of the ventricular wall occurs with cardiac chamber dilation, myocardial atrophy, aneurysms, syncope and ventricular arrhythmias that can lead to sudden death in young people and athletes [1–6]. Physical exercise and competitive sports activity may trigger life-threatening ventricular arrhythmias and accelerate the disease progression and the risk of sudden cardiac death (SCD) [7]. The estimated prevalence of AC in the general population ranges from 1:2000 to 1:5000 [5, 6, 8–10]. However, this frequency could be an underestimate due to difficulty or errors in diagnosis [11]. This pathology is worldwide distributed but in Italy, and especially in the Veneto region, the frequency is around 1:1000 [1, 12]. AC is a heterogeneous disease both clinically and genetically, and is mainly transmitted as an autosomal dominant form with incomplete penetrance [8], although there are recessive forms, such as Naxos and Carvajal syndromes [13, 14]. About 40% of AC cases carry one or more mutations in genes encoding for proteins of the intercellular junctional complexes known as desmosomes (i.e. plakophilin-2, PKP2 [15]; desmoplakin, DSP [16]; desmoglein-2, DSG2 [17]; desmocollin-2, DSC2 [18] and junctional plakoglobin, JUP) [19], which maintain the structural integrity of the myocardium [20–22]. Isolated reports, accounting for less than 1–3% of cases, are caused by variants in non-desmosomal genes [10]. DSP is the most abundant protein in desmosomes and mediates the binding between different junctional complexes and the cytoskeleton. It is composed of an N-terminal domain important for localization and interaction with other proteins such as PKP2, a central coiled-coil domain involved in protein dimerization, and a C-terminal domain that interacts directly with intermediate filaments. DSP is expressed in all desmosome-containing tissues [23], and alternative splicing of its messenger RNA (mRNA) produces two isoforms, which differ in the length of the central helical domains: DSP I, predominantly expressed in the heart and comprising 2871 amino acids, and DSP II, consisting of only 2271 amino acids. To date, more than 100 pathological variants of the *DSP* gene have been detected in 10-15% of AC cases [10, 24]. Despite the progress in identifying AC genes, the mechanism by which alterations in desmosomes cause fibro-fatty replacement of cardiomyocytes is not yet fully understood. Several studies on genetically modified cellular and animal models showed a connection between mutation in junctional/desmosomal proteins and the dysregulation of specific cell signalling pathways such as Wnt/β-catenin, TGF-β and Hippo/YAP-TAZ pathways [25–28]. More recently, zebrafish models have been involved in AC studies, with stimulating applications in mechanistic analyses and in vivo high throughput drug screening. Zebrafish is an excellent model organism to study human disease and identify novel therapeutic strategies [29, 30]. Asimaki and colleagues produced a Plakoglobin 2057del2 zebrafish model that allowed identifying SB216763, an agonist of Wnt/β-catenin pathway, as a suppressor of the AC phenotype, supporting that the downregulation of this pathway could be a mechanism involved in the determination of the disease [31]. Giuliodori and colleagues generated transient zebrafish models for the DSP-associated form (AC type 8) [32]. In that experimental setup, the progressive fibro-fatty replacement of the myocardium could not be observed within the short time window of antisense morpholino activity. Nevertheless, that study could allow a large screen of signalling pathways in vivo, identifying three dysregulated signals in AC (Wnt/β-catenin, TGF-β and Hippo/YAP-TAZ) of which Wnt was the most dramatically affected [32]. In this study, we have generated and characterized the first stable knock-out Dsp-associated AC zebrafish models, analyzable at larval and adult stages, to better understand the different steps involved in the onset and development of the disease. Moreover, we have investigated the effects of physical exercise in the exacerbation of the disease using mutant larvae and adults under physical effort. These approaches were combined with the drug modulation of Wnt/β-catenin signalling, extending its role as a final common pathway in the AC spectrum.

## Results

### Genotyping of *dspa* and *dspb* zebrafish mutants

The zebrafish genome contains two Desmoplakin genes, *dspa* (ZDB-GENE-030131-2743) and *dspb* (ZDB-GENE-030131-1662), each orthologous to the single human gene *DSP*. Both zebrafish loci were thus considered in this study. The genotyping of the *dspa* point mutation (C > T) in the *sa13356* line was performed through direct Sanger sequencing. The 13-bp deletion in the *dspb* mutant line was detected by 3.5% agarose gel electrophoresis of the PCR product, and confirmed by sequencing (Supplementary Fig. 1A–F).

### Genetic lesion of both Dsp genes reduces zebrafish larval survival

For each developmental stage, larvae from inter-crossed *dspa/dspb* lines were genotyped and their frequencies were normalized to the total population. All genotype frequencies follow the Mendel’s laws prediction; however, a survival reduction of borderline significance is observed for the double homozygous line (-aabb) (Supplementary Table 1).

### Genetic lesion of zebrafish Dsp genes reduce Dsp mRNA and protein levels

A quantitative real-time PCR was carried out on mRNA obtained from 3 dpf (days post fertilization) larvae, showing a decrease of *dspa* and *dspb* mRNA expression in *dspa* and *dspb* mutants (Supplementary Fig. 2A). After Western-Blot analysis, wild-type (WT) larvae displayed three bands of nearly 240 kDa; one for *dspa* and two for *dspb*. In the *dspa*^*+/−*^*; dpsb*^*+/−*^ (hereinafter named “-ab”) larvae, all Desmoplakin isoforms were present, although with a reduction of about half (60%) of their expression (alpha-tubulin was used for normalization). Interestingly, in *dspa* and *dspb* homozygous mutants (“-aa” and “-bb”), where the mutated isoforms were absent, the normal ones appeared anyway affected, with Dspa showing a 70% reduction in –bb, and Dspb a 30% reduction in -aa (Supplementary Fig. 2B; Supplementary File-Western blot for the original images).

### Zebrafish Dsp mutant embryos display cardiac abnormalities and developmental delay

**Fig. 1:**
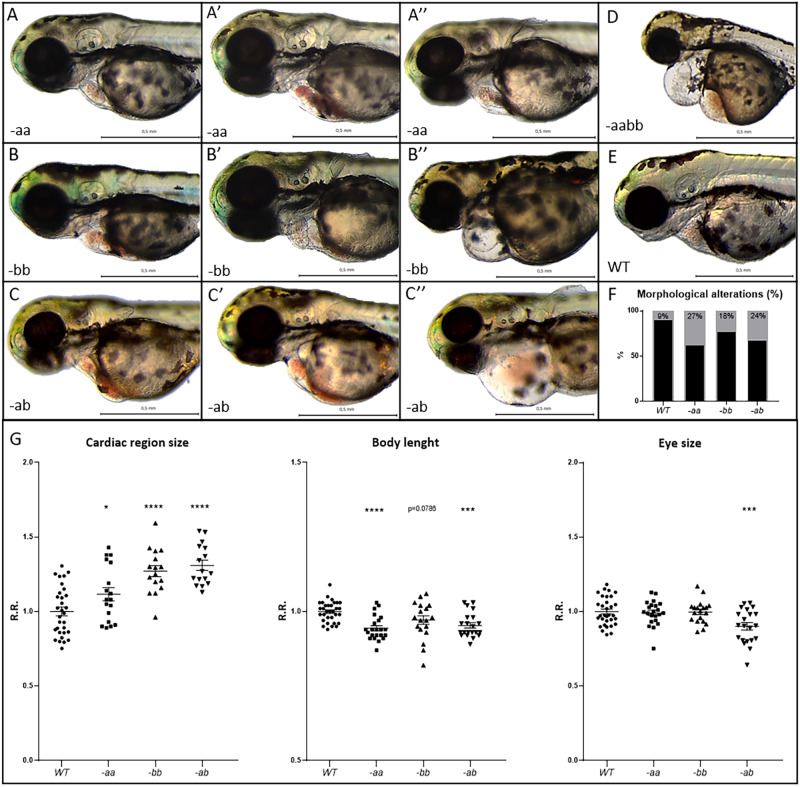
Cardiac alterations and developmental delay in Dsp mutant lines. **A**–**F** Mutant embryos (**A**–**C**″, **D**) display cardiac alterations, compared to WT (**E**). Homozygous -aa (**A**–**A**″) and -bb (**B**–**B**″) mutant hearts appear dilated and/or structurally altered, with cardiac pericardial effusion and/or hemopericardium in the cardiac region. Double heterozygotes -ab (**C**–**C**″) show a more serious phenotype than -aa and -bb lines. Double homozygotes -aabb display the most severe phenotype (**D**). **F** Percentage of heart alteration in different genotypes. Sample size: *n* = 100. **G** Cardiac size analysis shows that all mutants present heart dilation. Body length and eye size measurements indicate developmental delay in mutants, especially in the -ab condition. All embryos are at 3 dpf in lateral view, anterior to the left. Sample size Cardiac region size: WT *n* = 31; -aa n = 18; -bb n = 16; -ab *n* = 16. Sample size Body length: WT *n* = 32; -aa n = 22; -bb *n* = 19; -ab *n* = 21. Sample size Eye size: WT *n* = 32; -aa *n* = 22; -bb *n* = 19; -ab *n* = 21. R.R. relative ratio. Error bars: SEM. *p < 0.05; **p < 0.01; ***p < 0.001; ****p < 0.0001. Test: One-way ANOVA followed by Tukey’s test.

The percentage of embryos showing alterations of the cardiovascular system, clearly identifiable at 3 dpf, was significantly higher in Dsp mutants compared to WT controls. Specifically, the -ab and the -aa mutant lines showed phenotypic alteration in approximately 30% of analyzed animals (Fig. 1A–F). We noted that -aa and -bb mutant hearts resulted dilated and/or altered in the structure, with pericardial effusion and/or hemopericardium in the cardiac region. In some cases, accumulation or total absence of blood cells inside the heart was observed, suggesting alterations in the circulatory system, that in zebrafish embryos are anyway tolerated due to oxygen diffusion. These phenotypes appeared more serious in the -ab line, and extremely severe in the -aabb double homozygous condition (Fig. 1D). The analysis of the heart size showed a significant cardiac dilation in mutants (Fig. 1G). Finally, to evaluate if the cardiac phenotype was accompanied by a global developmental delay, we analyzed standard markers for growth retardation. All mutant conditions revealed a global reduction of body length and eye size, but only the -ab line showed a significant alteration of both parameters (Fig. 1G).

### Zebrafish Dsp mutant larvae display heart rate alterations

Heart rate analysis at embryonic/larval stages (Supplementary Fig. 3) revealed significant differences between WT and mutants (-aa, -bb, -ab), monitored at 2, 3, 5 and 7 dpf. In detail, the analysis at 5 and 7 dpf detected substantial bradycardia in all mutant genotypes (-aa, -bb, -ab). Considering the whole analysis, we can observe that the -ab condition was the first to show the most severe signs of bradycardia as early as 3 dpf, suggesting a faster worsening of heart condition compared to the other lines.

### Reduced survival of zebrafish Dsp mutants at juvenile stages

We performed specific crosses, generating WT, -aa, -bb, and -ab fish, to evaluate the survival rate of these genotypes at juvenile stages. We observed that all mutant fish, when compared to WT, showed a significant decrease of survival rate (Supplementary Fig. 4). The -aabb survival curve was not reported, due to the very low fitness and fertility of those individuals.

### Wnt/β-catenin and Hippo/YAP-TAZ signalling dysregulation in Dsp mutants

A quantitative real-time PCR analysis was performed in Dsp mutants to evaluate the activity of three signalling pathways, Wnt/β-catenin, Hippo/YAP-TAZ and TGF-β signalling, all previously correlated with AC [25, 28, 32]. We examined the expression of two Wnt/β-catenin signalling members, *ccnd1* and *myca*, confirming a downregulation of this pathway in 3 dpf mutants, although not always consistent for both markers in each genotype. Moreover, we detected general upregulation of YAP-TAZ signalling, evident for the *ccn2a* member in -aa mutants, and for *ccn2b* in all mutant lines (-aa, -bb, -ab) (Supplementary Fig. 5). To obtain tissue-specific resolution, these signalling cascades were also inspected taking advantage of pathway-specific transgenic lines. The Wnt reporter confirmed signal downregulation at the whole-body level, as well as in the cardiac region (data shown later). The Hippo/YAP-TAZ reporter showed instead comparable levels of signal activation at the whole-body level, but a cardiac-specific reduction in -ab mutants (Supplementary Fig. 6). Concerning the TGF-β signalling, both members considered for this pathway (*smad2* and *smad3*) showed a downregulation trend in all mutants (Supplementary Fig. 5).

### Choice of the double heterozygous Dsp mutant line as a model for AC

Based on the results obtained from the above-described experiments, we decided to focus our attention on the double heterozygous (-ab) mutant line as a suitable system to model the human genetic and phenotypic AC condition. This mutant line displayed a balanced reduction of both Dspa and Dspb protein expression, and showed a consistently severe phenotype in terms of cardiac morpho-function and signalling pathway activity. Thus, from now on, all reported experiments will refer to the -ab line.

### Detection of cardiovascular defects and inflammation in zebrafish Dsp mutants

**Fig. 2:**
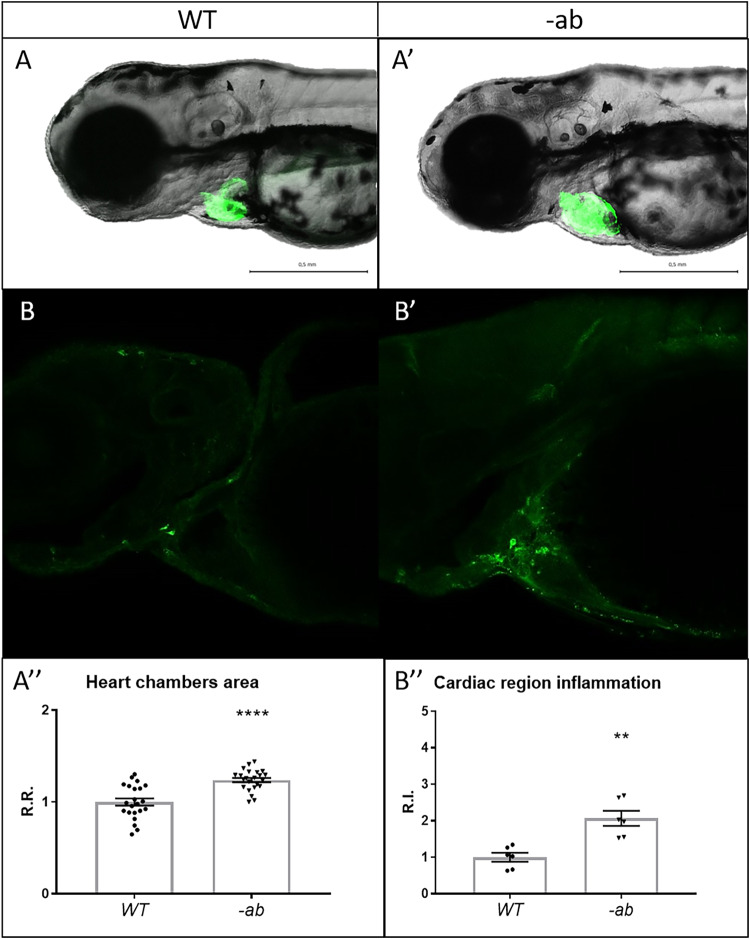
Detection of cardiac dilation and inflammation in zebrafish Dsp mutants. **A**–**A**′-**A**″ Heart morphology analysis of wild type and -ab mutant zebrafish embryos using a myocardial-specific transgene *Tg(tg:EGFP-myl7:EGFP)*^*ia300*^ identified a global dilation of both chambers in -ab mutants. All embryos are at 3 dpf, in lateral view. Sample size: *n* = 22 ± 1. R.R relative ratio. Error bars: SEM. ****p < 0.0001. Test: Unpaired t-test. **B**–**B**′–**B**″: Cells expressing the inflammatory marker L-plastin are more abundant in -ab mutants, compared to WT. All embryos are at 3 dpf, in lateral view, anterior to the left. Sample size: *n* = 6. R.I. relative intensity. Error bars: SEM. **p < 0.01. Test: Unpaired *t*-test.

The heart chambers structure was analyzed taking advantage of a myocardial-specific transgene *Tg(tg:EGFP-myl7:EGFP)*^*ia300*^. Mutated -ab embryos at 3 dpf showed an increase of the total area for both heart chambers (atrium and ventricle), indicating a prominent cardiac dilation (Fig. 2A–A′–A″). Moreover, reduced atrial and ventricular wall thickness was observed, as well as developmentally delayed atrioventricular valve, the latter characterized by less Wnt signalling expression in mutants, compared to wild-type controls (Supplementary Fig. 7). Cardiac size and function in -ab mutants were evaluated with the pyHeart4Fish imaging software, detecting increased cardiac size, atrium ejection fraction and atrial heartbeats. On the contrary, ejection fraction, relative contractility and heartbeats were diminished in the ventricle, suggesting impaired ventricular function, with potential compensation from the atrium (Supplementary Fig. 8, Supplementary Video 1 and 2). We failed to detect morphological defects in the vascular tree, observing, however, a reduced blood flow in -ab mutants, compared to controls (Supplementary Fig. 9, Supplementary Video 3 and 4).

To check if the observed alterations in mutant hearts could be accompanied by ongoing inflammatory processes, we used an anti L-plastin antibody as a leucocyte marker for the detection of inflammatory cells. This immunostaining revealed a two-fold increase of L-plastin positive cells in 3-dpf mutant hearts compared to controls (Fig. 2B–B′–B″). Other anatomical regions appeared similar between the two conditions, except for a three-fold increase of L-plastin positive cells detected in -ab mutants at the skin level, a well-known Desmoplakin-rich compartment (Supplementary Fig. 10).

### Impaired motor behaviour and exercise-induced mortality in zebrafish Dsp mutant larvae

**Fig. 3:**
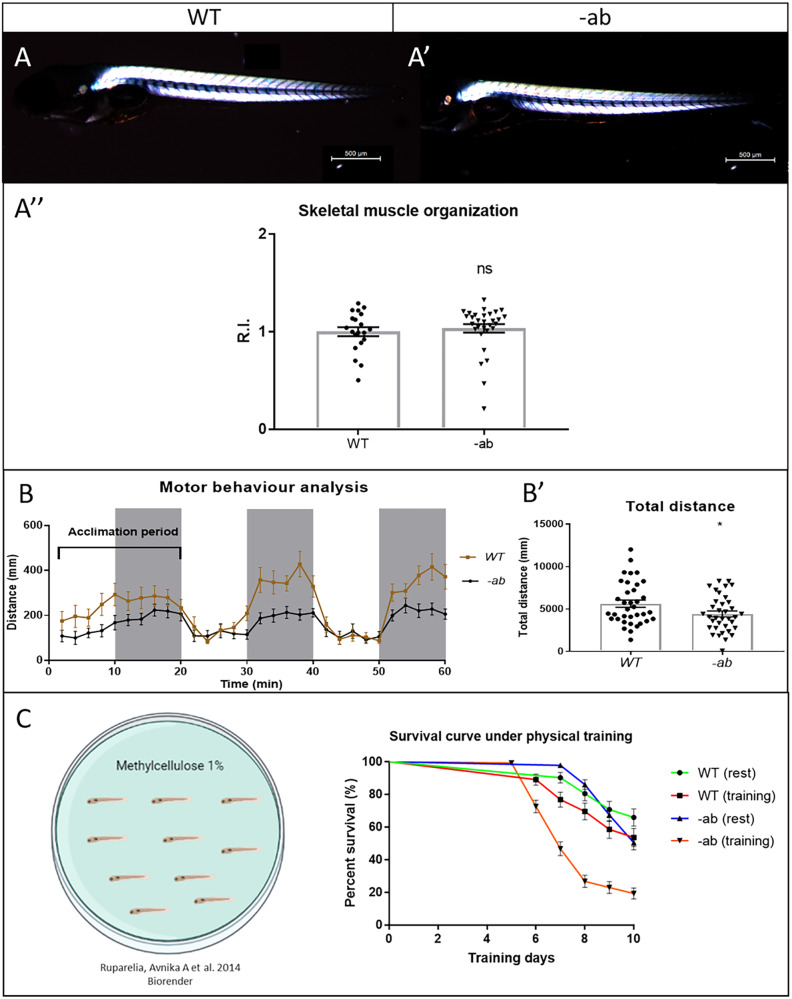
Impaired motor behaviour and exercise-induced mortality in zebrafish Dsp mutant larvae. **A**–**A**′–**A**″: Birefringence analysis on WT and mutant larvae at 5 dpf does not detect any significant difference in the skeletal muscles structure. All embryos are at 5 dpf, in lateral view, anterior to the left. Sample size: WT n = 20; -ab *n* = 30. R.I. relative intensity. Error bars: SEM. ns not significant. Test: Unpaired *t*-test. **B**–**B**′: WT and -ab larvae at 5 dpf display normal response to light (white areas) and dark (grey areas) stimuli, but with reduced motor performances if mutated, evaluated as total distance swum (**B**). Sample size: *n* = 36. Error bars: SEM. *p < 0.05. Test: Unpaired *t*-test. **C** A mild training protocol was applied on larvae from 3 to 13 dpf, for 10 days. A statistically significant increase in mortality was observed in trained mutants when compared with resting controls (p < 0.0001). Sample size: *n* = 50. Error bars: SEM. Test: Log-rank (Mantel–Cox) test.

Before subjecting the mutant lines to specific training protocols, we preliminarily checked the state of the skeletal musculature. To this purpose, we analyzed the skeletal muscle birefringence, known to be brighter in highly organized skeletal muscle fibres. In Fig. 3, we plotted the results of the analysis, performed on two pools of larvae, WT (Fig. 3A) and -ab (Fig. 3A′), represented as mean of birefringence intensity (from trunk skeletal muscle), normalized on the fish length. No differences were detected between the two pools (Fig. 3A″), indicating preserved organization of the skeletal muscle structure in 5-dpf Dsp mutants. Locomotor performances of 5-dpf larvae were imaged for 1 h under alternating light/dark periods. This test detected a normal response to light stimuli in mutants (Fig. 3B), but a significant reduction of the total distance swum (Fig. 3B′), compared to WT siblings, suggesting preserved sensory perception, but impaired motor performance. Since it is well known that the physical activity is a modulator involved in the exacerbation of AC, we decided to recreate this condition also in zebrafish larvae, by using a denser medium for their swimming (from 3 to 13 dpf, for 10 days). We observed an increased mortality in trained versus untrained pools. Also of note, Dsp mutants under exercise showed the highest mortality when compared with the other conditions (Fig. 3C).

### Downregulation of Wnt/β-catenin, Hippo/YAP-TAZ and TGF-β signalling in adult Dsp mutant hearts

**Fig. 4:**
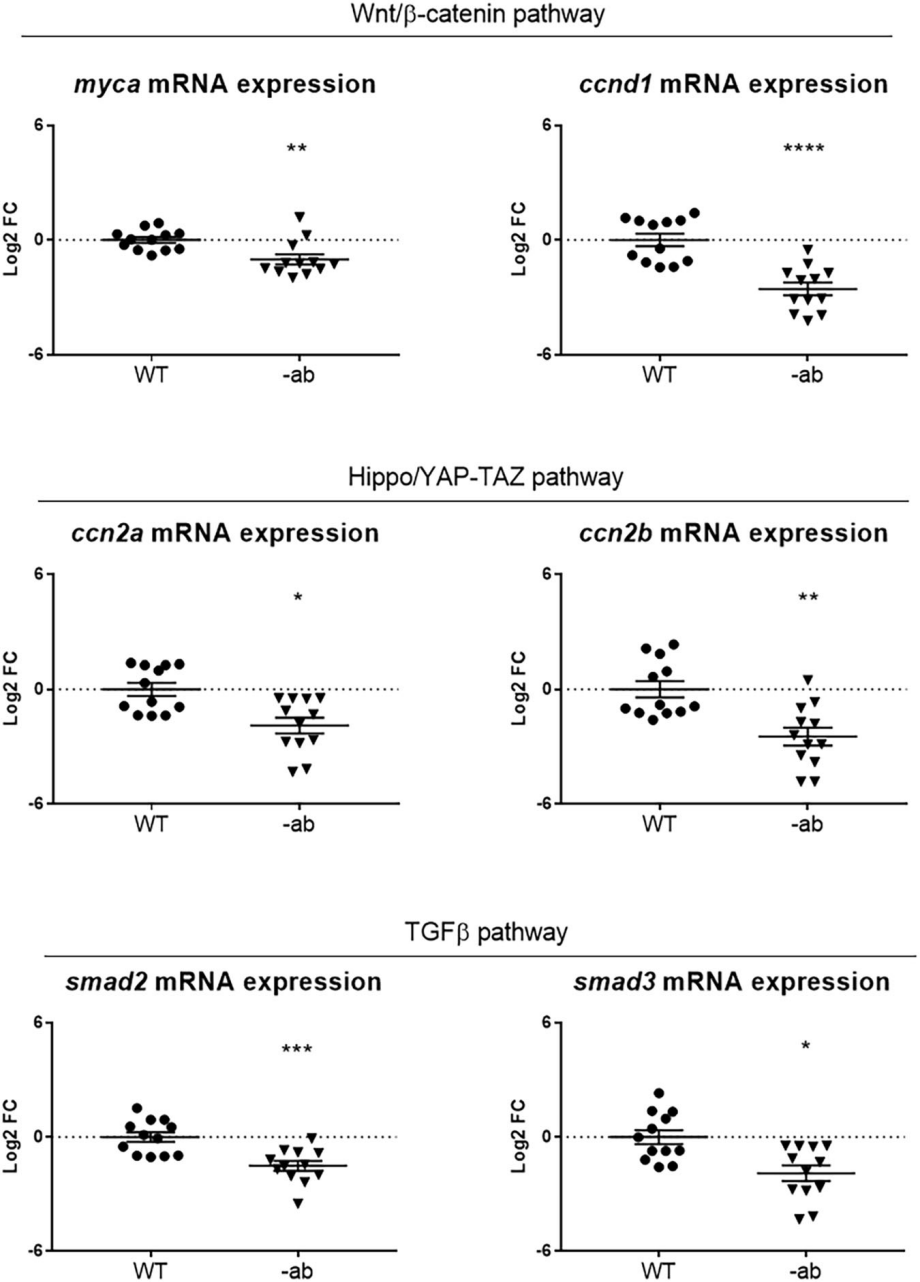
Signalling pathways dysregulation in Dsp mutant adult hearts. qPCR analysis of the expression of Wnt/β-catenin, YAP-TAZ and TGF-β signalling members showed a downregulation of all pathways in 1-year old Dsp mutant hearts. Each point on the graph corresponds to a pool of 3 hearts of the same genotype. Sample size: *n* = 12. Log2 FC: Log2 Fold Change. Error bars: SEM. *p < 0.05; **p < 0.01; ***p < 0.001; ****p < 0.0001. Test: Unpaired *t*-test.

We investigated possible dysregulation of Wnt/β-catenin, Hippo/YAP-TAZ and TGF-β signalling pathways in hearts of adult fish, making a comparison with the embryonic-larval condition. We examined the mRNA expression for Wnt/β-catenin signalling members *ccnd1* and *myca* in heart tissue from one-year old WT and -ab zebrafish, detecting a downregulation of this pathway in mutants, as observed at embryonic-larval stages (Fig. 4). We also checked the expression levels of members of Hippo/YAP-TAZ (*ccn2a* and *ccn2b*) and TGF-β (*smad2* and *smad3*) signalling, observing a downregulation of both pathways in Dsp mutant hearts (Fig. 4).

### Cardiac dilation and structural changes in Dsp mutant hearts are exacerbated by exercise

**Fig. 5:**
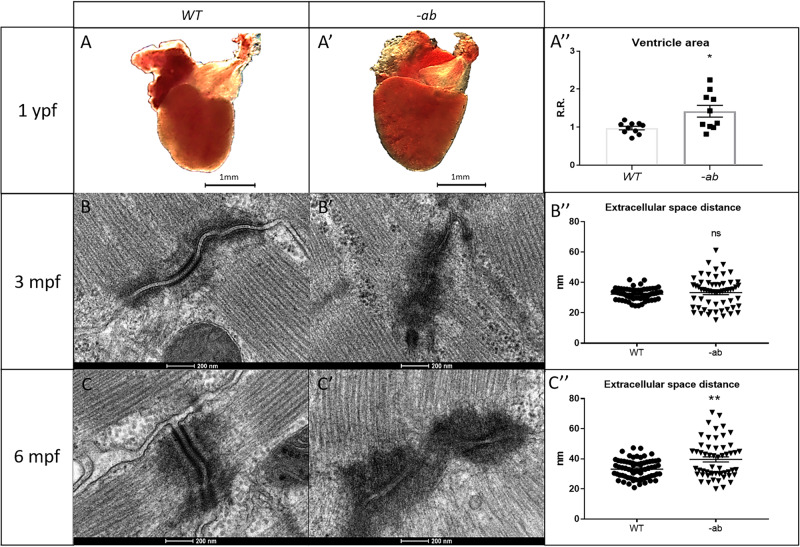
Cardiac dilation and TEM analysis of 3- and 6-month old Dsp mutant zebrafish hearts. **A**–**A**′-**A**″: 1-year old -ab mutant ventricles showed a statistically significantly dilation (*P* < 0.05) in comparison with WT controls. Sample size: *n* = 10. Error bars: SEM. R.R relative ratio. *p < 0.05. Test: Unpaired *t*-test. **B**–**B**′–**B**″–**C**–**C**′–**C**″: TEM analysis of 3- and 6-month-old mutated zebrafish heart revealed “pale”, disorganized and delocalized desmosomes. 6-month old -ab hearts showed a significantly increased distance in the extracellular space. Sample size: *n* = 60 ± 10 desmosome in 3 mutated and WT hearts. Error bars: SEM. ns not significant; **p < 0.01. Test: Unpaired t-test.

**Fig. 6:**
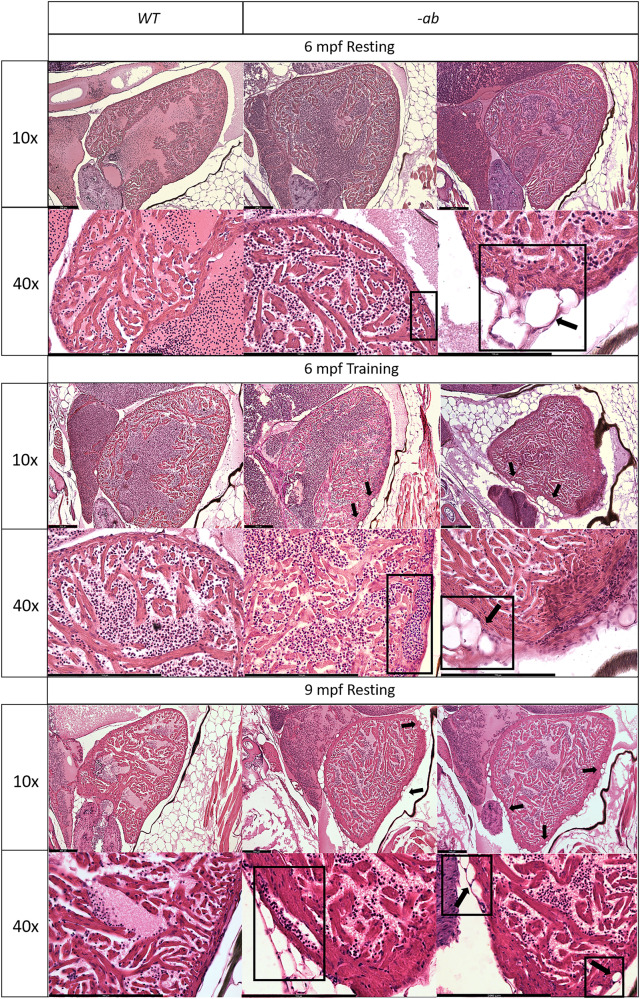
Cardiac dilation and structural changes in Dsp mutant hearts. Histological analysis of 6-month old -ab mutant zebrafish showed mild rarefaction of cardiomyocytes, thinning of the myocardial layer, age-related alterations in the distribution and organization of the trabeculae network, an abnormal shape of the ventricle, the presence of possible vessels dilation (rectangular box) and accumulation of adipose cells outside and inside the myocardial layer, in ≈50% of analyzed fish (square boxes and black arrows). Histological analysis of 6-month old mutated zebrafish confirmed the worsening of the condition after intensive physical training, like vessels dilation (rectangular box) and a more intrusive presence of adipose cells, in ≈80% of analyzed fish (square boxes and black arrows), showing similarities with 9-month old mutant hearts at rest. 9-month-old -ab mutant zebrafish showed worsening of the cardiac phenotype compared to 6-month old mutant hearts, with thickness of the myocardial layer, vessels dilation (rectangular box), and more intrusive presence of adipose cells, in ≈80% of analyzed fish (square boxes and black arrows). Sample size: *n* = 3 for each condition and age. Scale bar: 200 μm.

We performed the analysis of the ventricle area in adult hearts at 3, 6, and 12 months post fertilization (mpf), normalized to body size, detecting a significant (*p* < 0.5) cardiac dilatation in 1-year old ab mutants (Fig. 5A–A′–A″). This phenotype was not detectable at 3 and 6 mpf (Supplementary Fig. 11); however, a Transmission Electron Microscopy (TEM) analysis of 3 and 6 mpf samples revealed “pale” and disorganized desmosomes in mutant hearts (Fig. 5B′–C′ and Supplementary Fig. 12) when compared with WT (Fig. 5B–C), with an increased variability of the extracellular space distance (Fig. 5B″–C″), significant at 6 mpf. Histological analysis revealed no differences between WT and mutant heart at 3 mpf (data not shown). Instead, at 6 mpf, mutated zebrafish showed mild rarefaction of cardiomyocytes, thinning of the myocardial layer, age-related alterations in the distribution and organization of the trabeculae network, and an abnormal shape of the ventricle. Moreover, this analysis underlined the presence of vessels dilation and accumulation of adipose cells outside and inside the myocardial layer (Fig. 6, 6 mpf Resting). Fibrotic substitution was not observed (Supplementary Fig. 13). Of note, after the application of a training protocol from 3 to 6 mpf, the WT adult zebrafish hearts did not show any changes in tissue structure and organization, while the mutants showed an increased disorganization of the trabeculae, vessels dilations and presence of adipocytes within the myocardial layer (Fig. 6, 6 mpf Training). After doubling the duration of the training, no worsening of the condition was observed compared to WT (data not shown). At 9 mpf, all phenotypic anomalies observed at 6 mpf were confirmed, with a global worsening of the condition. The shape of the ventricle appeared more altered, with a trabeculae network disorganization. The myocardial layer appeared thicker, suggesting a possible compensatory process due to the poor contractility of the heart tissue. Moreover, in the myocardial layer the vessels appeared more dilated and the presence of adipose cells was more intrusive (Fig. 6, 9 mpf Resting), strengthening the idea that this disease is progressive in zebrafish as in humans.

### Wnt/β-catenin activation rescues AC phenotypes in zebrafish Dsp mutants

**Fig. 7:**
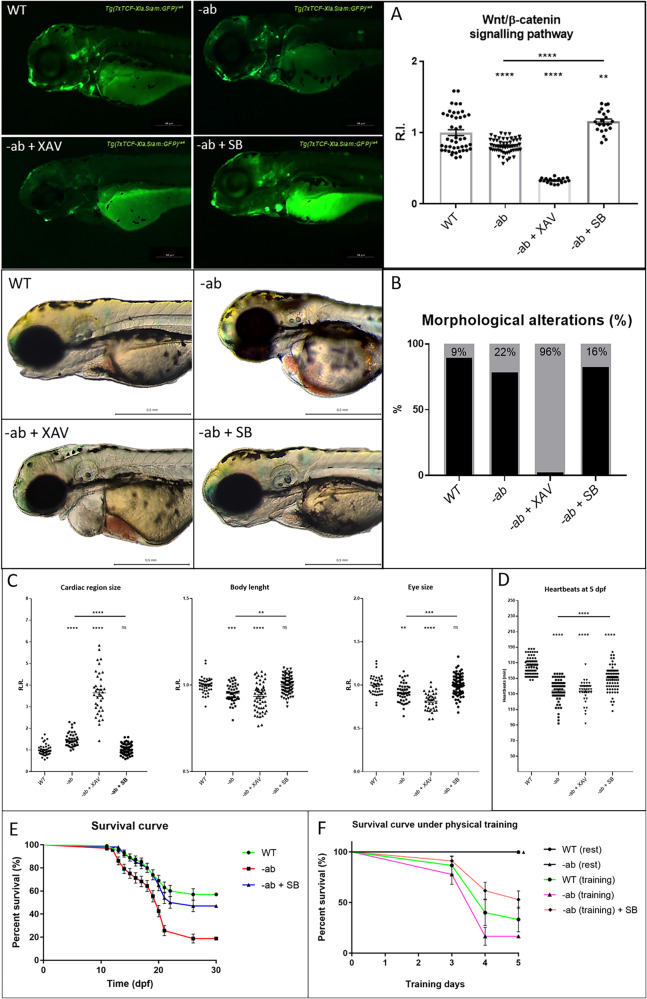
Wnt/β-catenin activation rescues AC phenotypes in Dsp zebrafish mutants. **A** -ab mutant zebrafish larvae in Wnt/β-catenin *Tg(7xTCF-Xla.Siam:EGFP)*^*ia4*^ transgenic background, treated for 2 days with 5 µM XAV939 (XAV, Wnt inhibitor) or 40 µM SB216763 (SB, Wnt agonist), showed a downregulation or a full recovery of the pathway activity (GFP reporter) when compared to WT controls or untreated mutants. All embryos are at 3 dpf and displayed in lateral view, anterior to the left. Sample size: WT *n* = 45; -ab *n* = 55; -ab + XAV *n* = 17; -ab + SB *n* = 21. R.I. relative intensity. Error bars: SEM. **p < 0.01; ****p < 0.0001. Test: One-way ANOVA followed by Tukey’s test. **B** The -ab larvae treated with 40 µM SB showed a significant reduction of cardiac dysmorphisms, while 5 µM XAV induced a worsening of the condition when compared to WT controls or untreated mutants. All embryos are at 3 dpf and displayed in lateral view, anterior to the left. Sample size: *n* = 100. **C** The -ab larvae treated for 2 days with 40 µM SB216763 showed a significant rescue of the main morphological alterations. The treatment with 5 µM XAV939 induced a worsening of the condition. Sample size Cardiac region size: WT *n* = 41; -ab *n* = 41; -ab + XAV *n* = 37; -ab + SB *n* = 82. Sample size Body length WT n = 41; -ab *n* = 46; -ab + XAV *n* = 54; -ab + SB *n* = 82. Sample size Eye size: WT *n* = 41; -ab *n* = 46; -ab + XAV *n* = 36; -ab + SB *n* = 81. R.R. relative ratio. Error bars: SEM. ns not significant; **p < 0.01; ***p < 0.001; ****p < 0.0001. Test: One-way ANOVA followed by Tukey’s test. **D** At 5 dpf, treated -ab larvae showed a statistically significant recovery of the bradycardia phenotype. Sample size: WT *n* = 70; -ab *n* = 66; -ab + XAV n = 40; -ab + SB *n* = 77. Error bars: SEM. ****p < 0.0001. Test: One-way ANOVA followed by Tukey’s test. **E** One-month survival rate of WT and -ab larvae untreated or treated with 40 µM SB216763 for 4 days; a statistically significant decreased mortality in mutants is observed after treatment, compared to untreated mutants (*p* < 0.0001). Sample size: *n* = 100. Error bars: SEM. Test: Log-rank (Mantel-Cox) test. **F:** 5-day survival analysis, from 3 to 8 dpf, of untrained or trained WT and -ab, as well as -ab simultaneously SB-treated and trained (training induced in 1% methylcellulose). Trained -ab, treated with SB, showed a statistically significant decrease in mortality when compared with -ab subjected to training in the absence of SB (*p* < 0.01). Sample size: *n* = 50. Error bars: SEM. Test: Test: Log-rank (Mantel–Cox) test.

Wnt/β-catenin signalling downregulation has been demonstrated to be central in the pathogenesis of AC. Therefore, we decided to treat the -ab line with the Wnt agonist SB216763 (SB) or the Wnt inhibitor XAV939 (XAV), from 1 to 3 dpf, to check for possible recovery or worsening of the pathway expression. The Dsp mutants, bearing the Wnt/β-catenin *Tg(7xTCF-Xla.Siam:EGFP)ia4* [33] reporter, revealed a significant recovery of pathway activation after treatment with 40 µM SB, while 5 µM XAV caused a strong reduction of Wnt signalling, when compared with WT control or untreated -ab pool (Fig. 7A). Interestingly, we observed a reduction of cardiac abnormalities in mutant larvae after SB treatment (Fig. 7B). After a 2-day treatment with either the agonist or the inhibitor of the Wnt/β-catenin signalling pathway, we noted respectively a recovery or a worsening of pathological parameters, including body length and eye size, as well as the level of cardiac dilation with pericardial effusion (Fig. 7C). We tried to rescue also the bradycardic phenotype by Wnt modulation. In this case, the treatment was carried out from 3 to 5 dpf. After a 2-day treatment with 5 µM XAV, the cardiac phenotype did not change significantly. On the other hand, after a 40 µM SB treatment, the mutant larvae partially recovered the bradycardic phenotype (Fig. 7D). As far as concerns the survival rate, mutants treated with 40 µM SB for 4 days presented a decrease in mortality, becoming like the WT condition (Fig. 7E). Finally, we decided to evaluate the SB effects on mutant larvae after physical activity in viscous medium (5 days - from 3 to 8 dpf - in 1% methylcellulose). The survival curves in Fig. 7F confirm a net difference between resting and training conditions, with untrained pools that ended the analysis without experiencing any fatality (100% of survivors). The comparison among trained pools showed similar death trends, starting at 6 dpf (the third day of training) and continuing with increasing slopes in the following days. Interestingly, the trained WT pool (green line) displayed a survival rate constantly placed in between the mutants’ curves. This is a noteworthy fact, since it implies that the trained plus SB-treated mutant pool—which ended up with the highest number of survivors (53%)—manifested an overall resistance to the physical training, even better than the WT reference. Looking instead at the differences between trained mutants with or without treatment, we can see that the pairwise comparison was statistically relevant, with a final number of survivors that was more than two times higher in SB-treated mutants compared to the untreated ones.

## Discussion

### Zebrafish heterozygous *dspa/dspb* mutants as suitable models for AC progression and training-induced modulation

The human *DSP* gene, underlying AC type 8, has two orthologs in zebrafish: *dspa* and *dspb*. We thus focused our attention on three genotypes that could better model in zebrafish the human heterozygous condition (-aa, -bb and -ab lines). Although the double homozygous line -aabb seemed a very attractive model to recapitulate severe AC phenotypes, it showed a very low fitness and the few fish that reached adulthood died unexpectedly, making the production of larvae for further studies very difficult. Quantitative real-time PCR analysis in all zebrafish Dsp mutants revealed a downregulation of mutated *dsp* genes expression, probably due to Nonsense-Mediated mRNA Decay (NMD). Western-Blot analysis confirmed the absence of the truncated proteins in -aa and -bb homozygous lines; also of note, a reduction of the non-mutated forms was detected, suggesting interdependence between Dspa and Dspb isoforms. Interestingly, the double heterozygous (-ab) line showed a less severe mRNA downregulation, supporting the hypothesis that a balanced model potentially better represents the heterozygous human condition. At larval stages of development, we observed alterations in the cardiac region of all mutant lines, associated with growth retardation, heart rate alteration and increased mortality. Of note, the detection of the bradycardia condition confirmed the results obtained in other zebrafish models for AC, with transient knock-down of *jup*, *pkp2*, *dsc2*, and *dsp* genes [32, 34–36]. Interestingly, mice models with specific loss of DSP protein expression in the sinoatrial node pacemaker tissue showed bradycardia, also in the absence of cardiomyopathy [37], suggesting a possible involvement of DSP in cardiac pacemaker function, with similar roles also in the zebrafish pacemaker [38]. Concerning the reduced viability observed in zebrafish Dsp (-aa, -bb, -ab) mutants, the –ab line showed the lowest percentage of survival, around 20%, emphasizing that this genotype appears the most severe and prone to larval death. Of note, cardiac alterations accounted for about 30% of the offspring, resembling the disease penetrance observed in humans [10]. Overall, the above observations point to zebrafish Dsp mutants, and especially the -ab line, as suitable systems to model Dsp-linked AC in terms of genotype-phenotype spectrum. The -ab genotype, implemented with a myocardial-specific fluorescent transgene, allowed to detect specific alterations in heart shape, including chamber dilation, a phenotypic condition described also in a recent DSP mouse model and in human patients [39, 40]. The presence of undergoing inflammatory processes in the cardiac region of -ab larvae was suggested by detecting an increased number of L-Plastin-positive cells in mutant hearts, compared to controls. The pan-leucocyte marker L-Plastin is in fact an actin-binding protein that plays a role in the activation of T-cells in response to co-stimulation through TCR/CD3 and CD2 or CD28, modulating also the cell surface expression of IL2RA/CD25 and CD69. At the larval stages considered in this study, the labelled leucocytes belong to the innate immunity (with major components being represented by macrophages and neutrophils), since adaptive immunity cells (e.g.: B and T lymphocytes) appear in zebrafish from three weeks post fertilization onwards [41]. Obviously, the inflammatory phenotype of these cells should be further investigated, to assess the comparability with human AC, and to evaluate these mechanisms as potential therapeutic targets.

Alterations in the motor behaviour and the impact of physical training on disease onset and progression were also inspected. Mutant -ab larvae showed a reduction in the normal motor activity; a birefringence analysis of the skeletal muscle excluded, however, potential fibre disorganization, pointing to other causes, including heart failure. Mutant larvae were subjected to a training protocol, which led to a strong increase in the mortality of trained mutants when compared to both resting mutants and wild-type controls. These results suggest that the lack of Dsp in these larvae caused an instability in heart structure/function under stress conditions, leading to premature death events.

Morphological analyses and endurance tests were performed on adult -ab mutant fish. The earliest phenotype, represented by desmosome defects, was detected by TEM at 3 mpf (although not corroborated by in vivo traction force analyses), followed by cardiomyocyte rarefaction at 6 mpf, dilated vessels and presence of adipocytes at 9 mpf, evidenced by H&E, and cardiac dilation, overtly observable in 1-year old hearts. Overall, these features faithfully recapitulate in zebrafish the progressive heart degeneration seen in AC patients and mice models, pointing at zebrafish as a suitable system to elucidate how these phenotypic conditions could temporally appear and mechanistically interact with each other.

Interestingly, following a training protocol, we observed phenotypic similarities between 6 mpf trained fish and 9 mpf untrained ones, corroborating the idea that an intensive training protocol can exacerbate/anticipate the cardiac condition, like in humans. On the other hand, three additional months of training did not induce a worsening of the alterations, suggesting the reaching of maximum damage and/or the activation of regeneration processes [42] counteracting further degeneration in the zebrafish heart.

### Wnt/β-catenin, Hippo/YAP-TAZ and TGF-β as AC common pathways in zebrafish Dsp models

Considering previous evidence of Wnt/β-catenin, Hippo/YAP-TAZ and TGF-β signalling dysregulation in AC, we examined these pathways in the zebrafish Dsp mutants. The expression analysis at larval stage of two Wnt/ β-catenin signalling members (*ccnd1* and *myca*) and a GFP-based Wnt biosensor indicated a downregulation of this pathway, at both cardiac and whole-body level, as previously shown for different AC models [32]. Hippo/YAP-TAZ signalling showed instead slight upregulation at the whole-body level, according to the analyzed pathway members (*ccn2a* and *ccn2b*), but a cardiac-specific downregulation, when examined with a GFP-based Hippo/YAP-TAZ reporter. TGF-β did not show any alteration at larval stage, suggesting that species-, stage- or genotype-specific differences may account for such finding. The three pathways were analyzed also at the adult stage; Wnt/β-catenin and Hippo/YAP-TAZ signalling confirmed their cardiac-specific downregulation under Dsp deficiency, like at the larval stages. TGF-β signalling was also found down-regulated in mutant hearts (where we failed to detect fibrosis), suggesting dynamic changes of this pathways under AC-like conditions, as previously suggested [32], due to complex tissue/stage/context-specific regulations, as well as repair processes and/or signalling compensations occurring in zebrafish [42].

### Modification of AC phenotypes by Wnt/β-catenin signalling modulation

Wnt/β-catenin signalling can be modulated by a wide range of drugs acting as agonists or antagonists for this pathway, including the inhibitor XAV939 or the activator SB216763, the latter identified by Asimaki and colleagues as a potential therapeutic molecule in a Plakoglobin-linked AC zebrafish model [32]. Moreover, it was demonstrated in a DSP mouse model that Wnt/β-catenin signalling regulators, AKT1 and GSK3-β, are dysregulated after physical exercise, validating across different species the central role of Wnt/β-catenin modulation in AC pathogenesis [43]. Our group has previously demonstrated the rescuing effects of SB216763 in transient (morpholino-induced) zebrafish model of Dsp-linked AC [32]. In this work, we evaluated the pharmacological effects of this drug in a stable Dsp mutant; the treatment with SB216763 partially rescued in -ab mutant larvae the survival rate, developmental parameters such as body length and eye size, and, interestingly, also the bradycardic phenotype. Of note, the mortality rate, observed after methylcellulose-induced training, was also rescued after drug exposure. Overall, these results, by demonstrating the rescuing effects of SB216763 in a stable Dsp mutant, point to Wnt/β-catenin signalling modulation as a promising therapeutic strategy for different forms of the AC spectrum, especially in counteracting exercise-induced effects. Moreover, the detection of Hippo/YAP-TAZ and TGF-β signalling dysregulation in this model confirms a robust molecular signature in AC, opening the way to a combination therapy targeting multiple pathways in AC.

## Methods

### Zebrafish maintenance

All experiments were performed in accordance with the Italian and European Legislations (Directive 2010/63/EU) and with permission for animal experimentation from the Ethics Committee of the University of Padova (OPBA) and the Italian Ministry of Health (Authorization number 407/2015-PR). *Danio rerio* (zebrafish) were kept in a temperature-controlled (28.5 °C) environment, with a 12:12 light-dark cycle, staged and maintained following standard procedures. Wild-type lines used in this work, also in the generation of the stable dsp mutant lines, included Tuebingen, Giotto and Umbria strains [44]. The following transgenic lines were used: Wnt/β-catenin *Tg(7xTCF-Xla.Siam:EGFP)ia4* [33] and Hippo/YAP-TAZ *Tg(Has.CTGF:EGFP)ia48* [45] reporter lines and the myocardial-specific line *Tg(tg:EGFP-myl7:EGFP)ia300* [46]. The transgenic and reporter lines, with and without mutated genetic background, were outcrossed with wild-type fish, to obtain a heterozygous fluorescent signal in all analyzed embryos.

### Generation and genetic analysis of *dspa* and *dspb* mutant lines

The zebrafish mutant line for desmoplakin a (*dspa*) was obtained from the Zebrafish Mutation Project (ZMP). The identifying allele *sa13356* carries a nonsense mutation (Chr 2: g.2324300C>T) in exon 23, leading to the formation of a premature stop codon (p.Gln960Ter). The zebrafish mutant line for desmoplakin b (*dspb*), bearing a 13-nucleotide deletion (Chr 20: g.52912248_52912260del), was produced in our laboratory (allele *ia303*) by using a CRISPR-Cas9 approach. To delete the functional protein, a single guide RNA (sgRNA) was designed using the CHOPCHOP software (https://chopchop.cbu.uib.no/), to specifically target an optimal CRISPR sequence in exon 3 of dspb gene, generated according to Gagnon and colleagues and transcribed in vitro using the MEGAshortscript T7 kit (AM1354, Life Technologies, Milan, Italy) (Supplementary Table 2) [47]. One-cell stage embryos were injected with 2 nL of a solution containing 280 ng/μL of Cas9 protein (M0646, New England Biolabs, Milan, Italy) and 100 ng/μL of sgRNA; phenol red was used as injection marker. F0 injected embryos were raised to adulthood and screened, by F1 genotyping, for germline transmission of the mutation. Heterozygous mutants, harbouring the mutation of choice, were outcrossed 4 times and then in-crossed to obtain homozygous mutants (F5 generation), stabilize the line and avoid CRISPR/Cas9-induced off-target effects. The WT fish used as controls in the experiments were “siblings”, originally obtained by outcrossing double heterozygotes.

### DNA extraction from zebrafish embryos and adults

The HotSHOT protocol [48] was used to prepare genomic DNA from whole embryos at 24–48 h post fertilization (hpf). Each embryo was placed in a tube with 0.16 mg/mL Tricaine solution in fish water. After 2 min, Tricaine was discarded and the tube was filled with 50 µL DNA lysis buffer (NaOH 50 µM). The tubes were heated at 95 °C for 20 min and cooled at 4 °C for 5 min; 5 µL of Tris HCl 1 M pH 7.5 were added to neutralize the DNA sample. Genomic DNA from zebrafish adults was prepared after caudal fin biopsy (fin clipping).

### Genotyping of *dspa* and *dspb* mutant lines

Homozygous and heterozygous nonsense mutations in *dspa* were identified by direct sequencing of *dspa*-specific PCR products. Homozygous and heterozygous 13-bp deletions in *dspb* were identified by *dspb*-specific PCR followed by agarose gel electrophoresis (3.5% p/v) (Supplementary Table 2).

### Body length and heart activities measurements

Fish lengths and heart rates were measured using a bright field microscope (Leica M165FC) equipped with a digital camera (Leica DFC7000T, Leica Microsystems, Milan, Italy), connected to a computer with Leica Software (LAS V4.8) for image acquisition and processing. The zebrafish were anesthetized with 0.16 mg/mL Tricaine solution in fish water to facilitate the measurement. Heart rates were determined by counting the number of atrial contractions during 60 s in embryos from 2 to 7 dpf. The number of heartbeats per unit of time was expressed as beats per minute (bpm).

Cardiac activities including atrial and ventricular ejection fraction, relative contractility and heartbeats were measured using the pyHeart4Fish imaging software [49].

For blood flow measurement, the following protocol was applied: automatic selection of the most variable regions in time (likely the regions where red blood cells are moving); calculation of the centreline for each selected segment; extraction of the grey levels along the centreline (low levels: red blood cells; high levels: plasma) for each frame; alignment of the successive centreline levels using dynamic time warping [50]; calculation of the speed at each point of the segment based on the alignment; calculation of the average speed along the segment; calculation of the average speed of the segments for each zebrafish.

### RNA isolation and quantitative real-time reverse transcription PCR (qRT-PCR)

For gene expression analysis, total RNA was extracted from pools of 30 3-dpf (days post fertilization) zebrafish embryos or from hearts of 12-month old zebrafish using TRIzol reagent (15596026, Thermo Fisher Scientific, Milan, Italy). One μg of total RNA was used for cDNA synthesis with M-MLV Reverse Transcriptase RNase H- (06-21-010000, Solis BioDyne, Tartu, Estonia), according to the manufacturer’s protocol. Quantitative PCR (qPCR) was performed in triplicate on batches of 3-dpf mutant larvae and pools of 1-year-ld isolated mutant hearts by using the 5X HOT FIREPolEvaGreen qPCR Mix Plus (Solis BioDyne) and Light Cycler 480 II (Roche, Basel, Switzerland), following the manufacturer’s protocol. All primers were designed using the software Primer3 (http://primer3.ut.ee), with optimal annealing temperature of 60 °C (Supplementary Table 2).

### Protein extraction

Pools of ten embryos were treated with 1 mL of deyolking buffer (1/2 Ginzburg Fish Ringer without Calcium: 55 mM NaCl, 1.8 mM KCl, 1.25 mM NaHCO3), shaken for 5 min at 1100 rpm to dissolve the yolk sac and centrifuged at 300 × *g* for 30 s to pellet the cells. Optionally, two additional wash steps were done by adding 1 mL of wash buffer (110 mM NaCl, 3.5 mM KCl, 2.7 mM CaCl_2_, 10 mM Tris/Cl pH 8.5), shaking 2 min at 1100 rpm and pelleting as before. Finally, pellets were dissolved in LDS-sample buffer and incubated for 5 min at 95 °C. Extracted proteins were frozen and conserved at −20 °C or directly loaded on a gel [51].

### Western blot

Proteins were separated on NuPAGE™ 3–8% Tris-Acetate Protein Gels, 1.5 mm, 10-well (Invitrogen, Milan, Italy). The Spectra Multicolor Broad Range Protein Ladder (Thermo Fisher Scientific) was used as molecular weight marker for protein samples in the 10–260 kDa range.

After the electrophoretic run, proteins were transferred from the gel to a PVDF (polyvinylidene difluoride) Immobilon-P transfer membrane by Mini gel tank apparatus (Invitrogen), following the manufacturer’s instructions. The membrane was incubated 1 h at 4 °C in 10% non-fat dry milk in TBS-T (0.1% Tween-20) to saturate, block and avoid non-specific binding of the antibody. Then, it was incubated overnight at 4°C with primary antibody diluted in 5% non-fat dry milk PBST. Secondary antibodies were incubated for 1 h in 5% non-fat dry milk TBS-T. The signal was detected using an Alliance 9 Mini Chemiluminescence Imaging System (Uvitec, Cambridge, UK), after incubation with Pierce™ ECL Western Blotting Substrate (Thermo Fisher Scientific). Band intensities were normalized against Tubulin protein expression. Primary antibodies used for Western blot were: α-Tubulin (mouse, A11126, Invitrogen) diluted 1:1000 in 5% non-fat dry milk TBST; Desmoplakin 1/2 (mouse, 651155, Progen, Heidelberg, Germany) diluted 1:200 in 5% non-fat dry milk TBST. Secondary antibodies used for Western Blot were: Anti-mouse IgG (goat) (H + L)-HRP Conjugate (Bio-Rad, Hercules, CA, USA) diluted 1:2000 in 5% non-fat dry milk TBST.

### Immunofluorescence

Embryos at 3 dpf were fixed in 4% PFA/PBS overnight at 4 °C and stored at −20 °C in 100% MetOH. Samples were rehydrated in MetOH/PBS series (75%, 50%, 25%), 5 min each, depigmented using 3% H2O2 and 1% KOH in PBS 1×, and washed 2 times in PBS 1X + 0.5% Triton X. Samples were washed in distilled water 5 min and frozen in acetone at −20 °C, 7 min, for tissue permeabilization. After additional washes for 5 min in distilled water and 5 min in PBS 1× + 0.5% Triton X, embryos were blocked 30 min in PBDT (PBS 1× + 1% BSA + 1% DMSO + 0.5% Triton X) plus 2% goat serum at RT, and then incubated for 2 days at 4°C in PBDT + 2% Goat serum + Rabbit Anti-Plastin L antibody (ab210099, Abcam, Cambridge, UK) 1:5000. After four washes for 15 min in PBDT, embryos were incubated in PBDT + Goat-anti-Rabbit AP (alkaline phosphatase) conjugated antibodies at 1:1000, overnight at 4 °C in the dark. Samples were washed four times for 15 min in PBDT and stained with 0.25 mg/mL Fast Blue BB (F3378, Sigma-Aldrich) + 0.25 mg/mL Naphthol-AS-MX-phosphate (N5000, Sigma-Aldrich) in staining buffer (100 mM Tris HCl pH 8.2, 50 mM MgCl_2_, 100 mM NaCl, 0.1% Tween 20). Finally, samples were stored in 4% PFA at 4 °C in the dark or embedded in 1.5% low melting agarose on a glass dish for acquisition with the confocal microscope (Leica SP5), exploiting the far-red emission from Fast Blue. The images were analyzed using ImageJ software.

### Transmission electron microscopy

Small pieces of heart tissue (about 2–3 mm^3^) were fixed with 2.5% glutaraldehyde (16220, EMS, Hatfield, PA, USA) plus 2% paraformaldehyde (P6148, Sigma-Aldrich) in 0.1 M sodium cacodylate buffer pH 7.4 overnight at 4 °C. Subsequently, the samples were post-fixed with 1% PFA in 0.1 M sodium cacodylate buffer for 1 h at 4 °C. After three water washes, samples were dehydrated in graded ethanol series and embedded in epoxy resin (46345, Sigma-Aldrich). Ultrathin sections (60–70 nm) were obtained with a Leica Ultracut EM UC7 ultramicrotome, counterstained with uranyl acetate and lead citrate and viewed with a Tecnai G2 transmission electron microscope (FEI, Hillsboro, OR, USA) operating at 100 kV. Images were captured with a Veleta digital camera (Olympus Soft Imaging System, Olympus, Segrate, Italy). At the desmosome level, the extracellular space distance was measured three times, in five different points, along each desmosome considered in the analysis (Supplementary Fig. 14).

### Histology

Three, six and twelve months old adult zebrafish were euthanized and fixed in Bouin solution (Picric acid in milliQ water/ Formalin/ glacial acetic acid) for a minimum of 24 up to a maximum of 72 h at room temperature, depending on the dimension of the fish. To allow a better fixation, the abdomen was opened along the ventral midline starting from the anal pore. The fixed samples were washed in ethanol 70% plus ammonia solution until the fish appeared with normal colour and the washing solution was clean. After dehydration in a series of graded ethanol and clearing by xylene, samples were embedded with paraffin. Paraffin sectioning (7–8 µm), hematoxylin & eosin (HE) and Masson trichrome staining were conducted based on standard procedures and photographed under a light microscope.

### Birefringence assay

Anesthetized embryos were placed in 2% methyl cellulose and subsequently placed on a glass slide. The specimen was then inserted between two polarizing-rotating filters and subsequently analyzed under a Leica M165FC microscope. Skeletal muscle fluorescence was recorded in bright field with a DFC7000T digital camera (Leica Microsystems), rotating the upper polarizing filter until the light refracted by the skeletal muscles was uniform and homogeneous all along the larval body. The pixel intensity was then analyzed with the ImageJ software.

### Locomotion assay

Behavioural experiments were performed using the DanioVision tracking system (Noldus Information Technology, Wageningen, The Netherlands). Zebrafish larvae at 5 dpf were placed in 48-well plates, with one larva per well in 1 mL of fish water. After 20 min of acclimation, movements of larvae were recorded repeating three cycles of 10 min of light and 10 min of dark, as previously described [52].

### Physical exercise of zebrafish larvae and adults

Wild type and mutated zebrafish larvae were let grow in 1% Methylcellulose solution dissolved in water. This solution, due to its high density, increased the force needed to reach food and move normally, simulating a physical training protocol [53] For mechanical stress induction in adults, groups of 3 mpf mutant and WT fish were put inside an in-house swim tunnel. The water flow was created using an electric pump with different power levels (Brushless DC Pump AD20P-1230D; Qmax = 67 cm^3^/s equal to 85 cm/s). In a natural environment, zebrafish swim with a speed between 3.5–13.9 cm/s [54] We divided the power of the electric pump into three different levels (1/2/3) where 1 corresponds to 11 cm/s, 2–12 cm/s and 3–13 cm/s output. We started with a training protocol characterized by a 1-h workout, including 5 min at power level 1 and 2 (acclimation), 50 min at level 3, representing the real intense workout, and 5 min at level 2 and 1, returning to rest. The training was repeated 5 days per week for 3 months; this protocol allowed recovery from manipulation stress and exercise during the 2 days of the weekend (Supplementary Fig. 15).

### Pharmacological modulation of the Wnt/β-catenin signalling pathway

Zebrafish embryos were treated for 48 h with 40 µM SB216763 or 5 µM XAV939 (S3442 and X3004, Sigma-Aldrich, Milan, Italy), directly dissolved in fish water, to activate (SB216763) or inhibit (XAV939) Wnt/β-catenin signalling.

### Signal quantification and statistical analysis

Signal quantification and morphological analysis were performed using the Measurements option of the Volocity 6.0 software (Perkin Elmer, Milan, Italy). Pairwise analysis was carried out by unpaired t-test. Multiple comparisons were performed by one-way ANOVA followed by Tukey’s test, while survival analysis was made using Log-rank (Mantel-Cox) test (Graph Pad Prism V7.0 software). The equality of the variances was analyzed by F test. In the charts, error bars display standard errors of the mean. Asterisks indicate significant differences from controls. Correspondence between asterisks and significance levels is indicated in the figure legends. Sample final sizes were obtained after collection and randomization from multiple mating events, excluding unfertilized eggs or embryos displaying very early developmental defects in all conditions. The sample size was preliminarily calculated by G*Power and Sample Size Calculator analysis. Single-blind experiments were performed at least in duplicate, with at least 10 processed individuals per condition, measured with at least two technical replicates and with at least three samples per condition acquired for imaging analysis.

### Supplementary information

Supplementary Material

Video 1

Video 2

Video 3

Video 4

Original Data File

## Data Availability

Data generated in the current study are available from the corresponding authors on reasonable request.
