# Supplementary material for: A novel DSP zebrafish model reveals training- and drug-induced modulation of arrhythmogenic cardiomyopathy phenotypes
Source: Cell Death Discov. 2023 Dec 6;9:441. doi: 10.1038/s41420-023-01741-2 (PMC10700616; doi:10.1038/s41420-023-01741-2)
Supplement: Supplementary file 1 — Supplementary Material [file 41420_2023_1741_MOESM1_ESM.docx]

**A novel DSP zebrafish model reveals training- and drug-induced modulation of arrhythmogenic cardiomyopathy phenotypes**

**Supplementary Data**

**Supplementary Tables**

|  | 3 dpf (n=187) | | 14 dpf (n= 160) | | Expected values | |
| --- | --- | --- | --- | --- | --- | --- |
| Genotype | N. | % | N. | % | Frequency | % |
| A.B. | 100 | 53.47 | 95 | 59.37 | 9/16 | 56.25 |
| A.bb | 32 | 17.11 | 34 | 21.25 | 3/16 | 18.75 |
| aaB. | 40 | 21.3 | 29 | 18.12 | 3/16 | 18.75 |
| aabb | 15 | 8 | 2 | 1.25 | 1/16 | 6.25 |
| Chi-square |  | 2.766 |  | 7.244 |  |  |
| p-value |  | 0.4292 |  | 0.0645 |  |  |

**Supplementary Table 1: Analysis of genotype frequencies from *dspa/dspb* inter-cross.**

Counting of the larvae, obtained from the inter-cross of double heterozygous (AaBb) fish, at 3 and 14 dpf. Number of individuals observed for each of the 4 pools of genotypes (columns "N.") and the relative frequency in percent (columns "%") are reported. The dot symbols (.) imply either wild type or mutated alleles for each gene. In the right columns, the expected frequencies and percentages are reported, based on Mendelian inheritance ("expected values "). The Chi-square statistical analysis shows that all genotype frequencies follow the Mendel’s law prediction; a reduction of the survival (p-value 0.0645) is observed for the aabb (double homozygous) individuals.

**A**

| Constant oligo | CRISPR/Cas9 DNA oligomer for gRNA | 5'-AAAAGCACCGACTCGGTGCCACTTTTTCAAGTTGATAACGGA  CTAGCCTTATTTTAACTTGCTATTTCTAGCTCTAAAAC-3' |
| --- | --- | --- |
| *dspb*-specific oligo | CRISPR/Cas9 DNA oligomer for gRNA  (gRNA sequence) | 5'-ATTTAGGTGACACTATAGGAAGTGCATCTCCAGACTGGTTT  TAGAGCTAGAAATAGCAAG-3' |

**B**

| Gene name | Forward primer  (5’-3’) | Reverse primer  (5’-3’) | Product  size (bp) |
| --- | --- | --- | --- |
| *dspa* | ATCGAGGAGGAAAAGCGCAA | GCCTCATCCTGCAGCTGTAA | 194 |
| *dspb* | CAAAATGGGCCCGGATGAG | AGCTTCTGGTCTTCGGCTC | 114 |

**C**

| Gene name | Signalling  Pathway | Forward primer  (5’-3’) | Reverse primer  (5’-3’) | Product  size (bp) |
| --- | --- | --- | --- | --- |
| *dspa* |  | ACATTCGCAACTCCATCACG | TCCAGAGCATGTAGTCCAGC | 156 |
| *dspb* |  | ATGCTGAACGAACTCAACGC | TATCTCTGCACTTCCTCCGG | 148 |
| *gapdh* | Housekeeping | GTGGAGTCTACTGGTGTCTTC | GTGCAGGAGGCATTGCTTACA | 173 |
| *ccnd1* | Wnt/β-catenin | CCAACTTCCTCTCGCAAGTC | TGGTCTCTGTGGAGATGTGC | 123 |
| *myc* | Wnt/β-catenin | AGAAAGCTGGAGTCCTCGAC | CTGCTGCAGTGTGTTCAGC | 118 |
| *smad2* | TGFβ | TCATGTCATCTACTGCCGCC | GTCTTGGCACGAGAACAGGA | 176 |
| *smad3* | TGFβ | CTATCAGCGGGTCGAGACAC | AGTTGCTCTGGGGTTCGATG | 149 |
| *ccn2a* | Hippo/YAP-TAZ | CTCCCCAAGTAACCGTCGTA | CTACAGCACCGTCCAGACAC | 140 |
| *ccn2b* | Hippo/YAP-TAZ | CCCACAAGAAGACACCTTCC | ATTCGCTCCATTCAGTGGTC | 119 |

**Supplementary Table 2: List of oligonucleotides for Dsp mutant generation and analysis.**

The table lists the sgRNA oligos for *dspb* mutant line generation (A), the primers for PCR-based genotyping (B), and for gene expression analysis by quantitative Real-Time RT-PCR (C).

**Supplementary Figures**


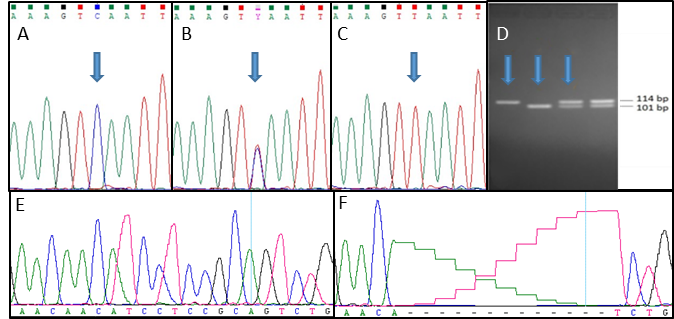


**Supplementary Figure 1**: **Genotyping of *dspa* and *dspb* zebrafish mutants.**

A-C: sequence-based identification of the *dspa* sa13356 point mutation (arrow) in case of homozygous control (A), heterozygous (B) and homozygous mutant (C). D: gel-based identification of the three *dspb* genotypes (arrows); from left to right: undeleted control, homozygous deletion and heterozygous condition. E-F: sequence-based identification of the *dspb* 13-bp deletion in homozygous mutant (F), compared to control (E).


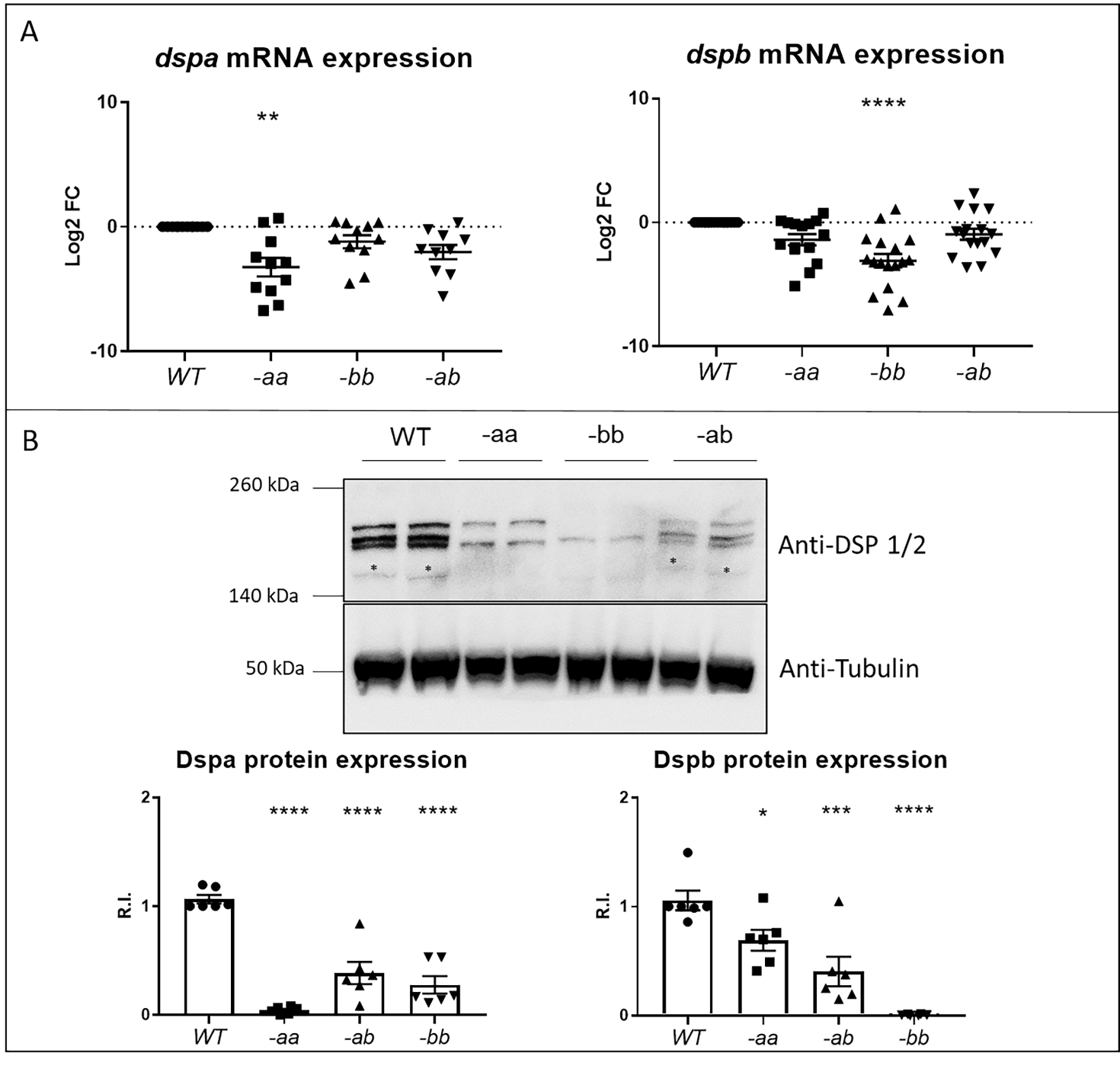


**Supplementary Figure 2: Reduced Dsp mRNA and protein levels in zebrafish Dsp mutants.**

A: qPCR analysis of *dspa* and *dspb* mRNAs expression in 3 dpf embryos of four different genotypes (WT: *dspa^+/+^; dspb^+/+^;* -aa: *dspa^-/-^; dspb^+/+^;* -bb: *dspa^+/+^; dspb^-/-^*; -ab: *dspa^+/-^; dspb ^+/-^*). The *dspa and dspb* mRNA expression decreases significantly in -aa and -bb, respectively. Each point on the graph corresponds to a pool of 30 embryos of the same genotype. Sample size: n= 11±5. Log2 FC: Log2 Fold Change. **=p<0.01; ****=p<0.0001. Error bars: SEM. Test: One-way ANOVA followed by Tukey’s test. B: Western blot analysis on 3 dpf embryos detected alterations in Dspa and Dspb protein expression. The WT sample presents 3 bands of nearly 240 kDa (A), one for Dspa and two for the Dspb isoforms. The -aa and -bb homozygous samples exhibit only the not mutated isoforms. In the -ab sample, all isoforms are present, with a reduced amount. Quantification of Desmoplakin levels detected a reduction to 52%, 23%, and 45% in -aa, -bb and -ab samples, respectively. Each point corresponds to a pool of 10 embryos of the same genotype. Asterisks in images indicate not specific signals. Sample size: n=6. R.I: Relative Intensity. *=p<0.05; ***=p<0.001; ****=p<0.0001. Error bars: SEM. Test: One-way ANOVA followed by Tukey’s test.


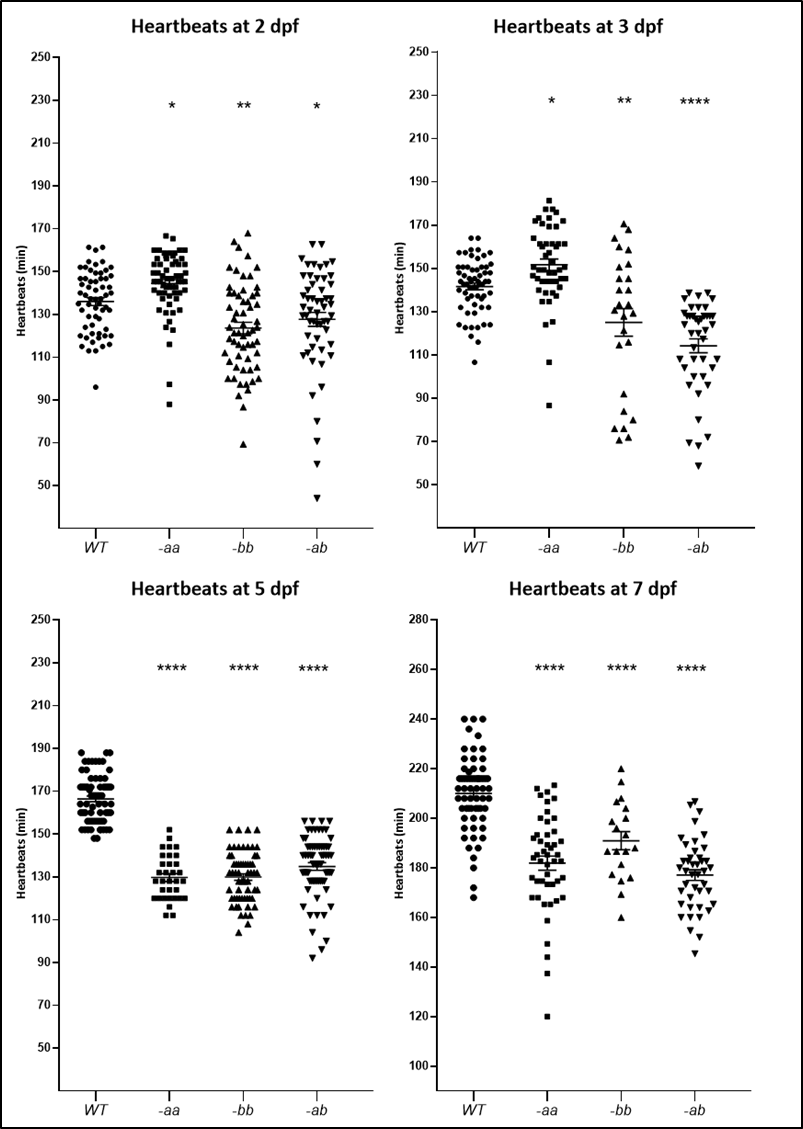


**Supplementary Figure 3: Heart rate alterations in Dsp mutant embryos and larvae.**

At 2 dpf, the heart rate frequency in WT was 136 beats per minute (bpm), while in -ab was 127 bpm; in -aa and -bb was 144 bpm and 123, respectively. At 3 dpf, heartbeat was 142 bpm in WT and 114 bpm in -ab; -aa and -bb presented 125 and 152 bpm, respectively. At 5 dpf, the heartbeat changes appeared more evident; -aa larvae display a significant bradycardia phenotype (130 bpm), like -ab (139 bpm) and -bb (130 bpm), compared to WT (166 bpm). At 7 dpf, the heart rate of mutant larvae was significantly reduced for all genotypes. Sample size 2 dpf: WT n=60; -aa n=60; -bb n=60; -ab n=55. Sample size 3 dpf: WT n=60; -aa n=48; -bb n=26; -ab n=42. Sample size 5 dpf: WT n=70; -aa n=33; -bb n=60; -ab n=66. Sample size 7 dpf: WT n=63; -aa n=47; -bb n=19; -ab n=42. *=p<0.05; **=p<0.01; ****=p<0.0001. Error bars: SEM. Test: One-way ANOVA followed by Tukey’s test.


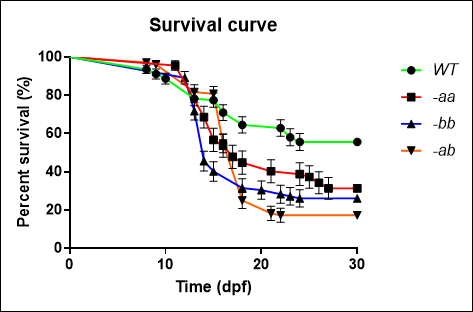


**Supplementary Figure 4: Decreased survival of zebrafish Dsp mutants at juvenile stages.**

One-month survival analysis of mutant lines showed a significant decrease of survival rate in mutated larvae (-aa (P<0.01), -bb (P< 0.0001), -ab (P< 0.0001)), compared to WT. The -aabb survival curve is not displayed due to the very low fitness and fertility of these individuals. Sample size: n=100. Error bars: SEM. Test: Log-rank (Mantel-Cox) test.


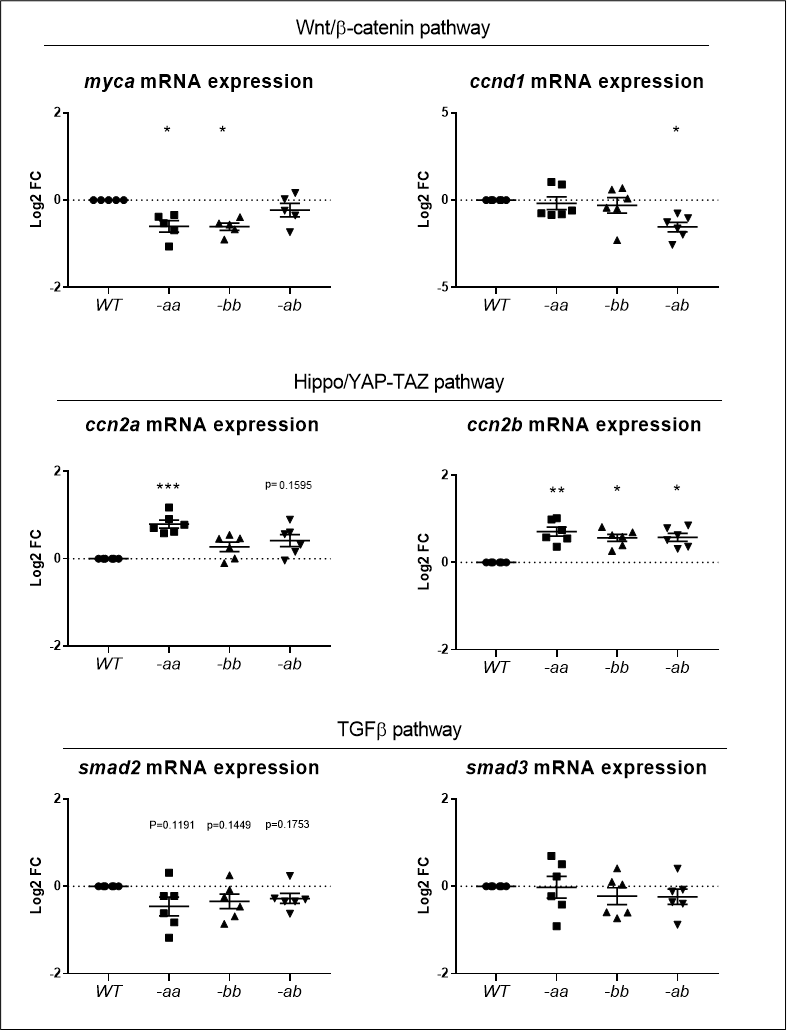


**Supplementary Figure 5: Signalling pathways dysregulation in Dsp mutant larvae.**

qPCR analysis detected Wnt/β-catenin, YAP-TAZ and TGF-β signaling dysregulation in Dsp mutants, significant for the first two pathways and as a trend for TGF-β. Each point on the graph corresponds to a pool of 30 embryos of the same genotype. Sample size: n=6±1. Log2 FC: Log2 Fold Change. *=p<0.05; **=p<0.01; ***=p<0.001. Error bars: SEM. Test: One-way ANOVA followed by Tukey’s test.


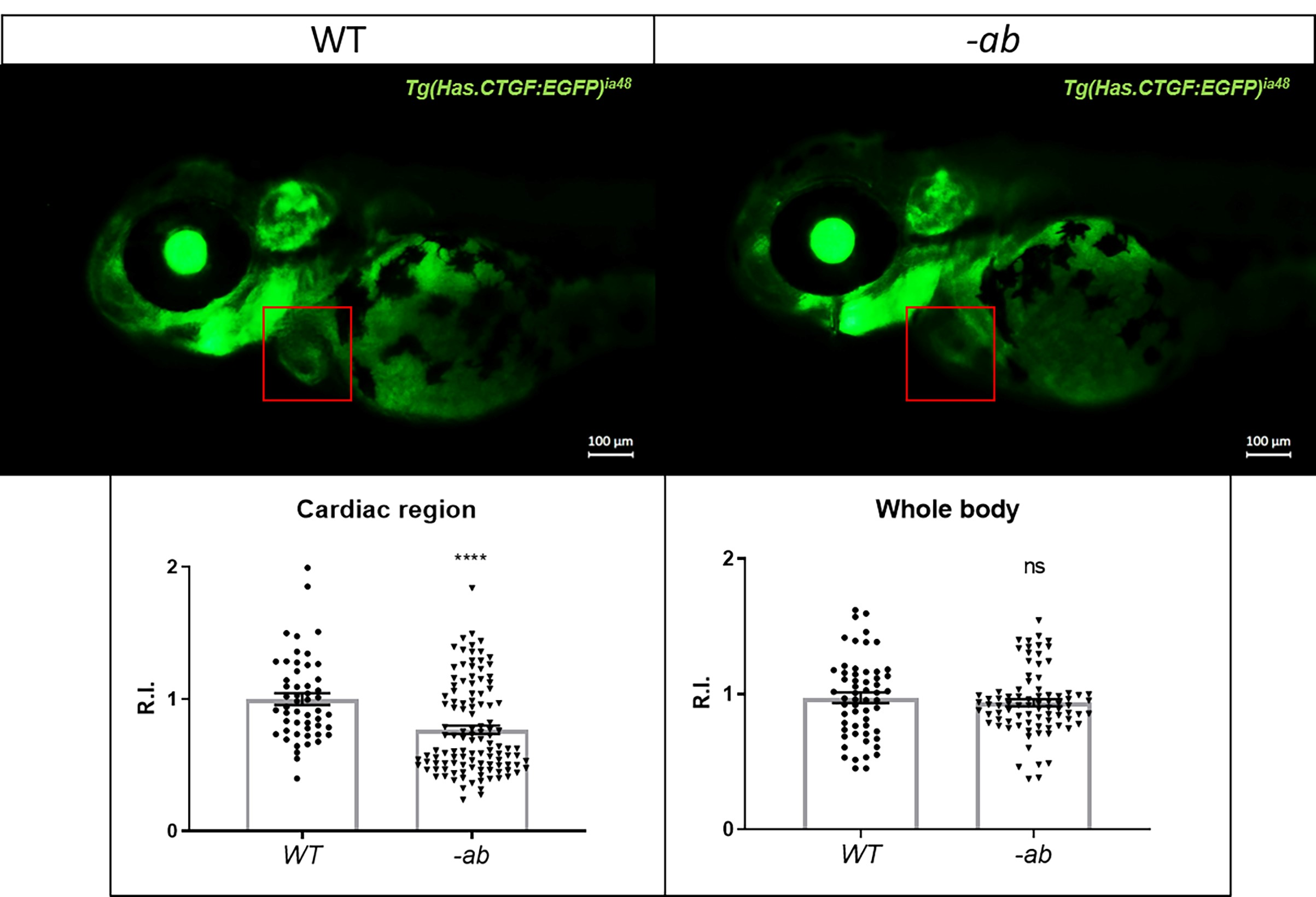


**Supplementary Figure 6: Hippo/YAP-TAZ signaling pathway expression in Dsp zebrafish mutants.**

Analysis of 3 dpf Hippo/YAP-TAZ specific *Tg(Has.CTGF:EGFP)^ia48^* transgenic mutant (-ab) larvae revealed a significant reduction of this pathway in the cardiac region of Dsp mutant larvae. The whole-body expression analysis of the pathway, instead, did not show any changes between mutants and controls. All embryos are at 3 dpf, in lateral view, anterior to the left. Sample size: WT n=50±10; *-ab* n=100±10. R.I.: Relative Intensity. Error bars: SEM. ns =not significant; ****=p<0.0001. Test: Unpaired t-test.

**Supplementary Figure 7: Abnormal atrial and ventricular wall structure and atrioventricular valve morphology in Dsp zebrafish mutants.**

Upper panel: Analysis of myocardium-specific *Tg(tg:EGFP-myl7:EGFP)^ia300^* transgenic mutant (*-ab*) larvae revealed a significant thinning of both ventricular and atrial myocardial tissue at 3 dpf. Sample size: n= 18. Scale bar: 20 μm; A: atrium; V: ventricle; avc: atrioventricular canal. Error bars: SEM. ****=p<0.0001. Test: Unpaired t-test. Lower panel: Analysis of 3 dpf Wnt/β-catenin *Tg(7xTCF-Xla.Siam:GFP)^ia4^* transgenic mutant (*-ab*) larvae detected developmentally delayed atrioventricular valves (avv), with reduced Wnt signaling expression. Sample size: n= 6. Scale bar: 20 μm. A: atrium; V: ventricle. R.I.: Relative Intensity. Error bars: SEM. *=p<0.05. Test: Unpaired t-test. All panels display cardiac regions at 3 dpf, in ventral view, anterior to the top.


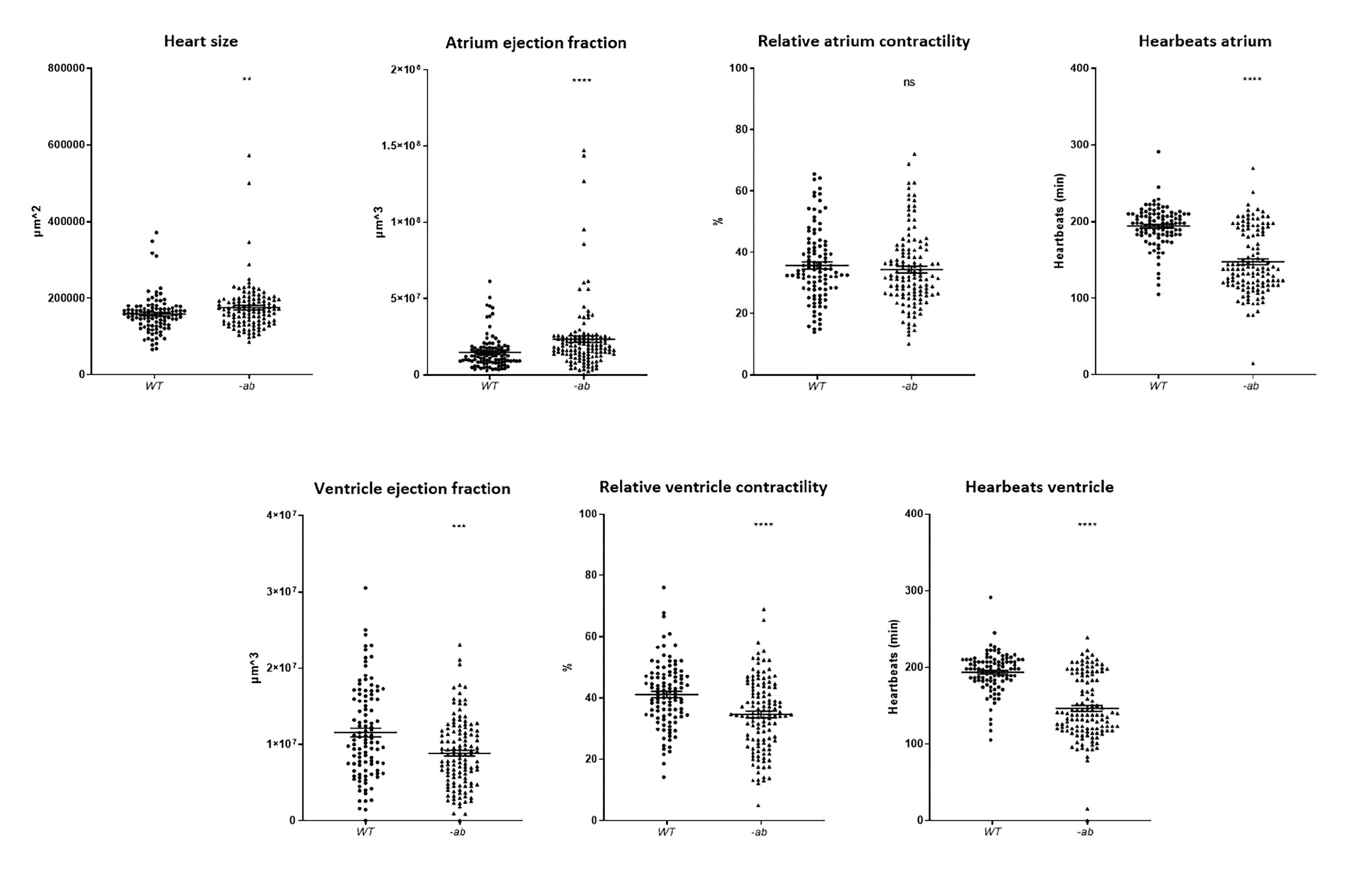


**Supplementary Figure 8: Heart chambers activity changes in Dsp zebrafish mutants.**

A significant dilation of the heart chambers was observed in -ab mutants compared to wild type siblings. Atrium contractility appeared not affected, presenting a conserved/increased ejection fraction. The ventricle displayed a lower ejection fraction, with a 20% reduced contractility. Both chambers presented bradycardia. All embryos were recorded at 3 dpf, in ventral view. Sample size: n= 100. Error bars: SEM. ns =not significant; **=p<0.01; ***=p<0.001; ****=p<0.0001. Test: Unpaired t-test.


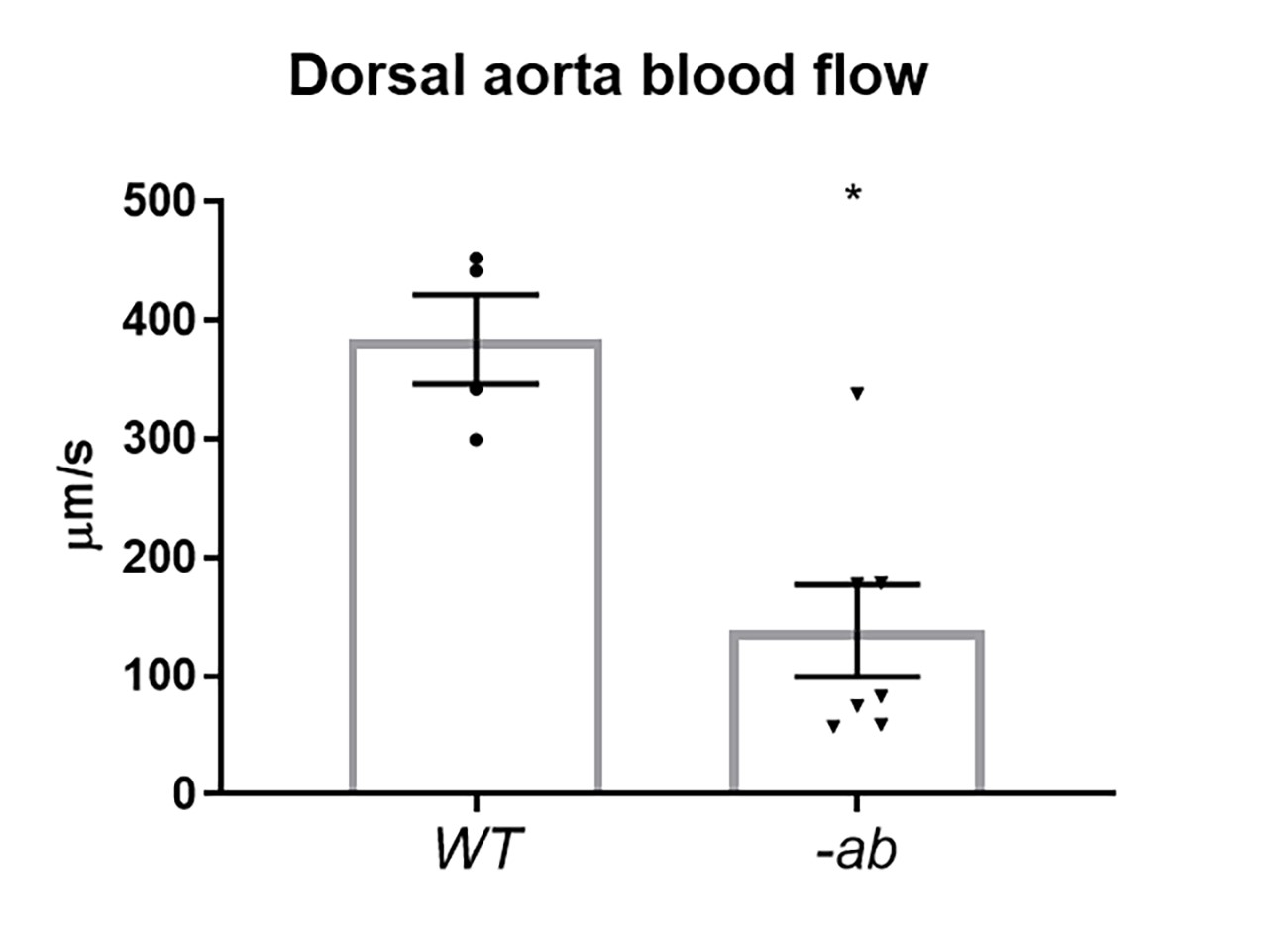


**Supplementary Figure 9: Blood flow velocity in dorsal aorta of Dsp zebrafish mutants.**

The measurement of the movement of blood cells in dorsal aorta showed a decreased blood flow in mutant (-ab) larvae, compared to wild type controls. All embryos were recorded at 3 dpf, in lateral view. Sample size: WT n= 4; -ab n= 7. Error bars: SEM. *=p<0.05. Test: Unpaired t-test.


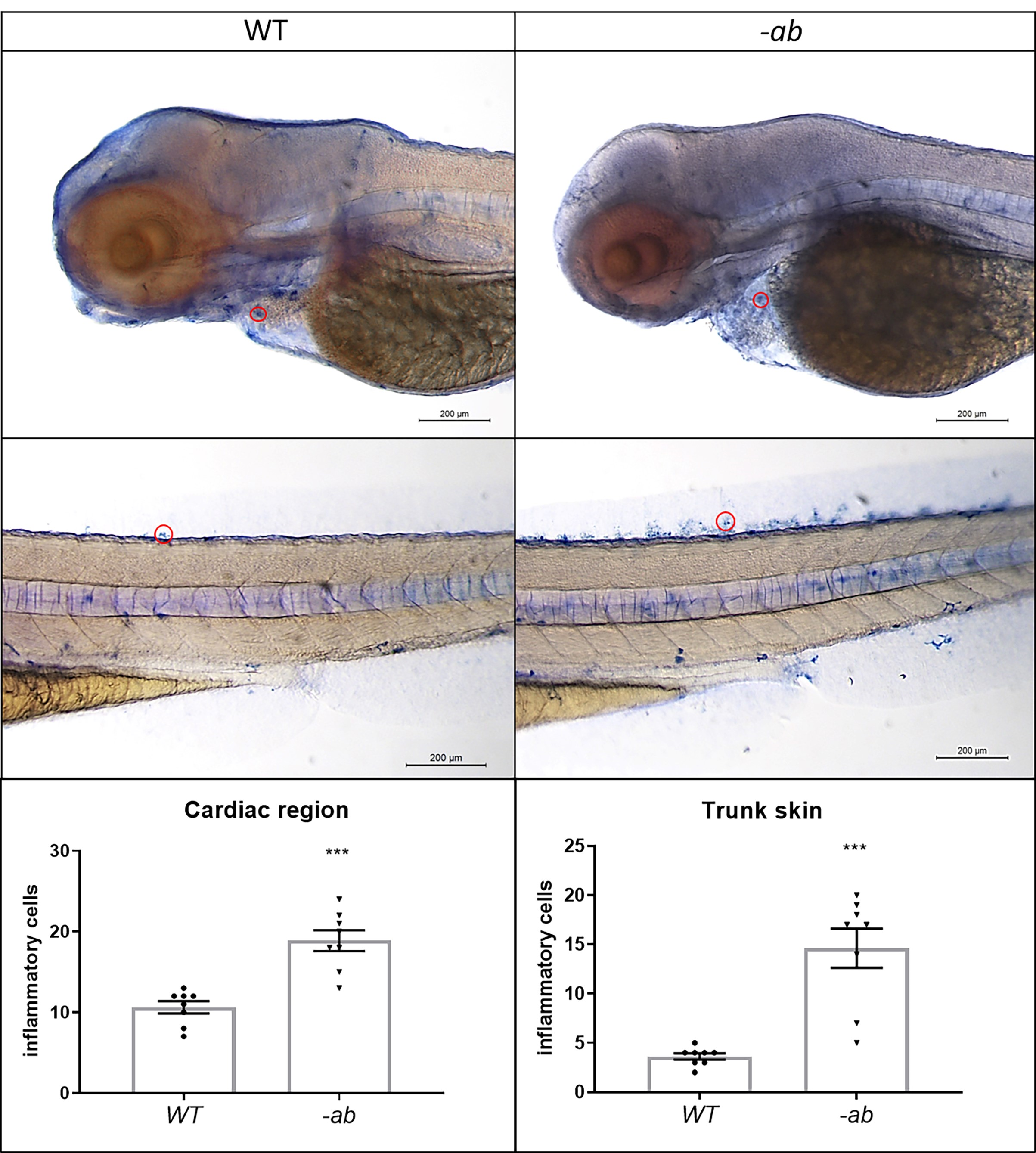


**Supplementary Figure 10: L-plastin positive cells localization in Dsp zebrafish cardiac region and skin.**

Cells expressing the inflammatory marker L-plastin (representative examples in red circles) are more abundant in the cardiac region and skin of -ab mutants, compared to WT controls. All embryos are at 3 dpf, in lateral view, anterior to the left. Sample size: n=8. Error bars: SEM. ***=p<0.001. Test: Unpaired t-test.

**
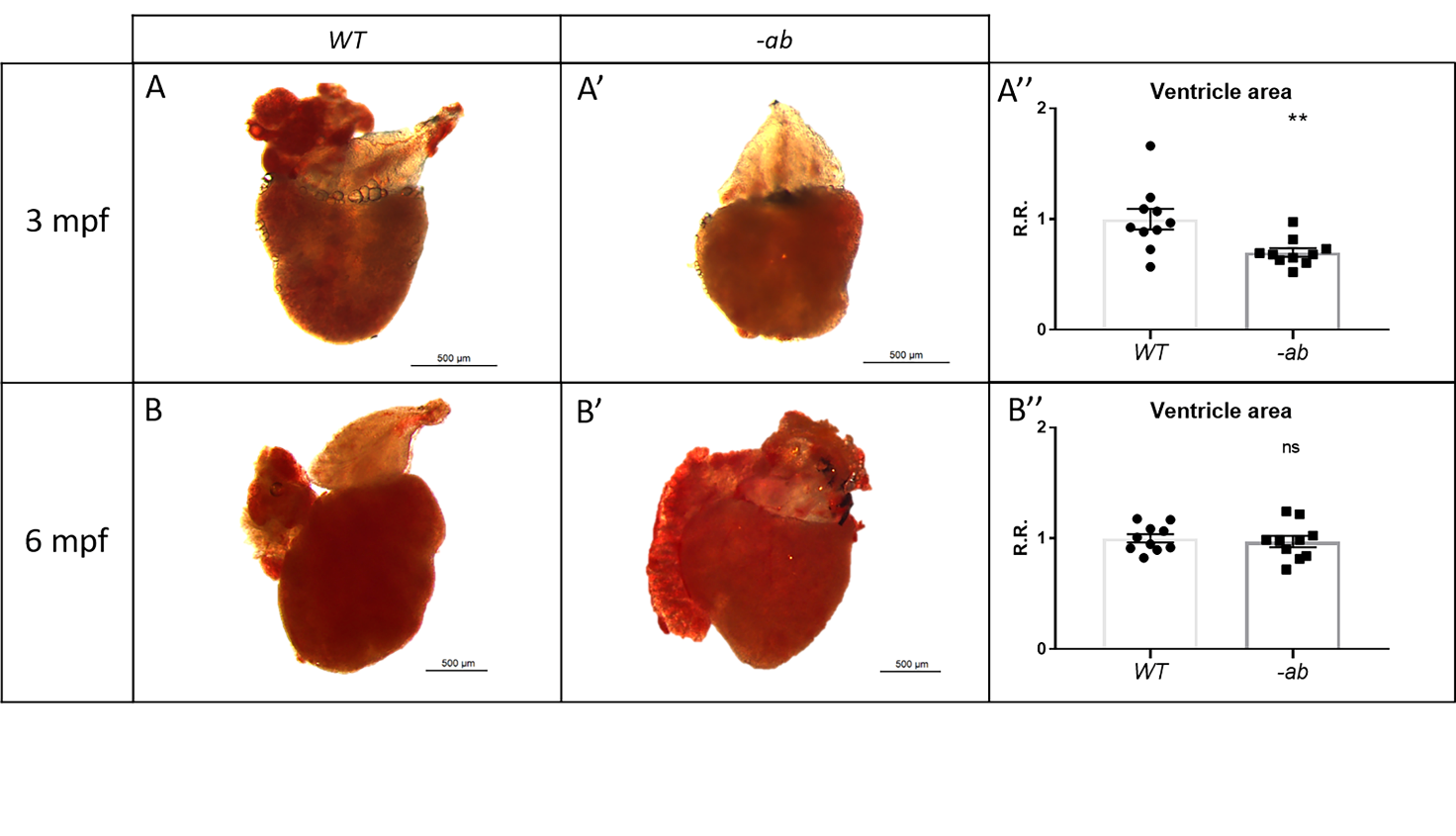
**

**Supplementary Figure 11: Cardiac dilation in Dsp mutant hearts.**

A-A’-A’’: 3 months old -ab mutant ventricles showed a statistically significantly reduction (P<0.01) in comparison with WT controls. Sample size: n= 10. R.R: Relative Ratio. **=p<0.01. Test: Unpaired t-test.

B-B’-B’’: 6 months old -ab mutant ventricles did not show dilation (ns) in comparison with WT controls. Sample size: n= 10. R.R: Relative Ratio. ns= not significant. Error bars: SEM. Test: Unpaired t-test.


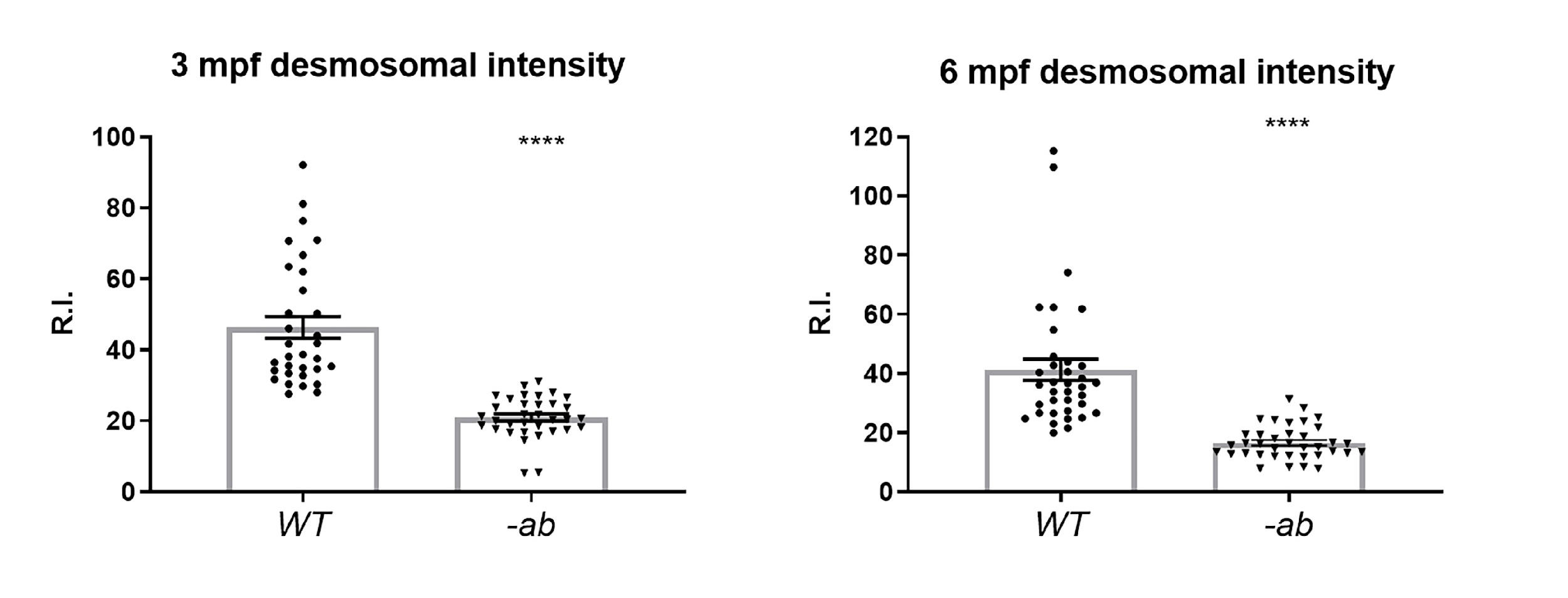


**Supplementary Figure 12: Desmosomal intracellular plaque signal analysis in Dsp mutants.**

Desmosomal intracellular plaque signal analysis of 3- and 6-month old mutated zebrafish heart revealed a “pale” desmosome phenotype, with a significantly reduced signal in that region. Sample size: n= 30±10 desmosome in 3 mutated and WT hearts. Error bars: SEM. ***=p<0.001. Test: Unpaired t-test.


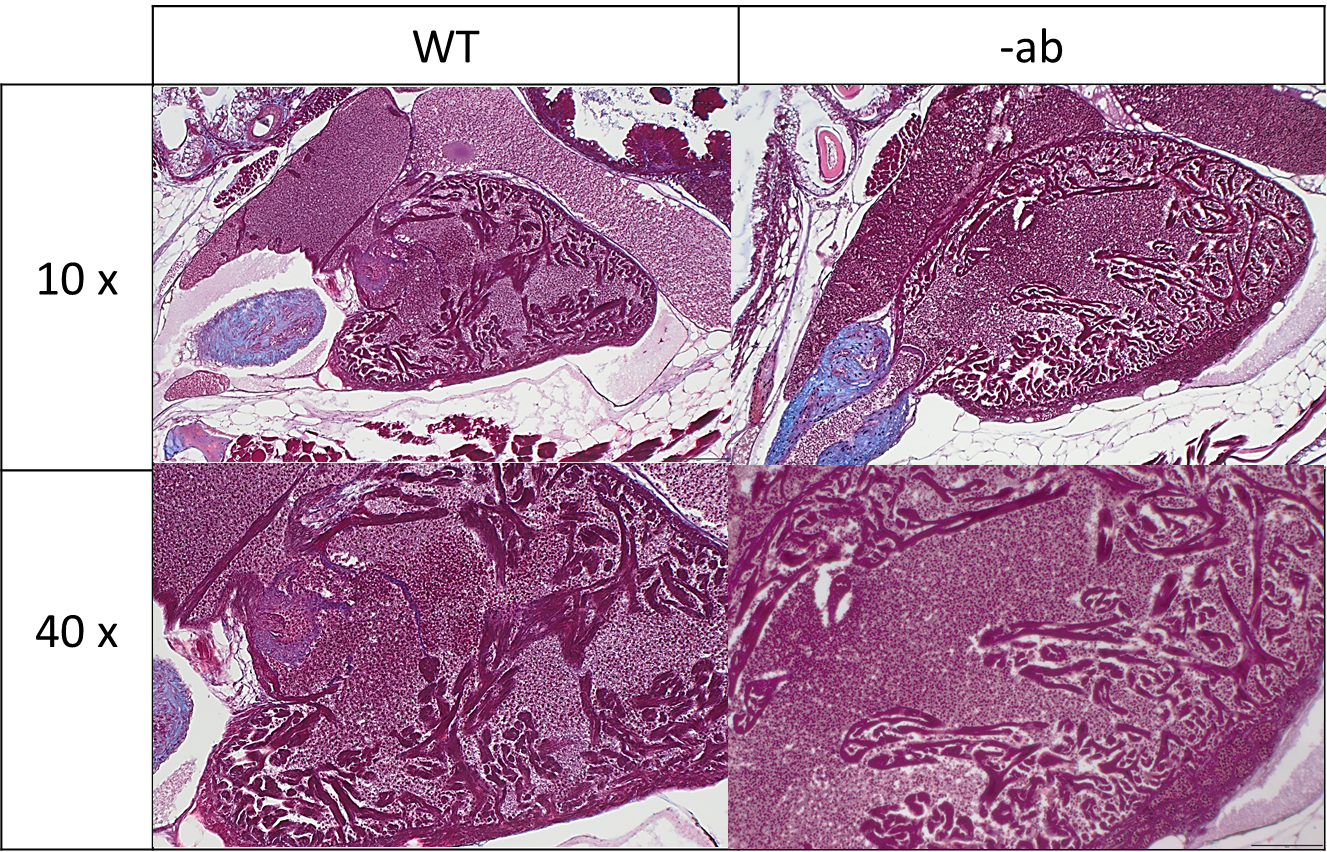


**Supplementary Figure 13: Masson trichrome staining analysis in Dsp mutant hearts.**

Masson trichrome staining analysis of 9-month old -ab mutant zebrafish showed no signs of fibrotic substitution in the myocardial layer of both wild type and –ab mutated zebrafish hearts. The expected blue staining in the outflow tract validated the success of the methodology. Sample size: n=3 for each condition. Scale bar: 200 μm in both 10X and 40X magnifications.


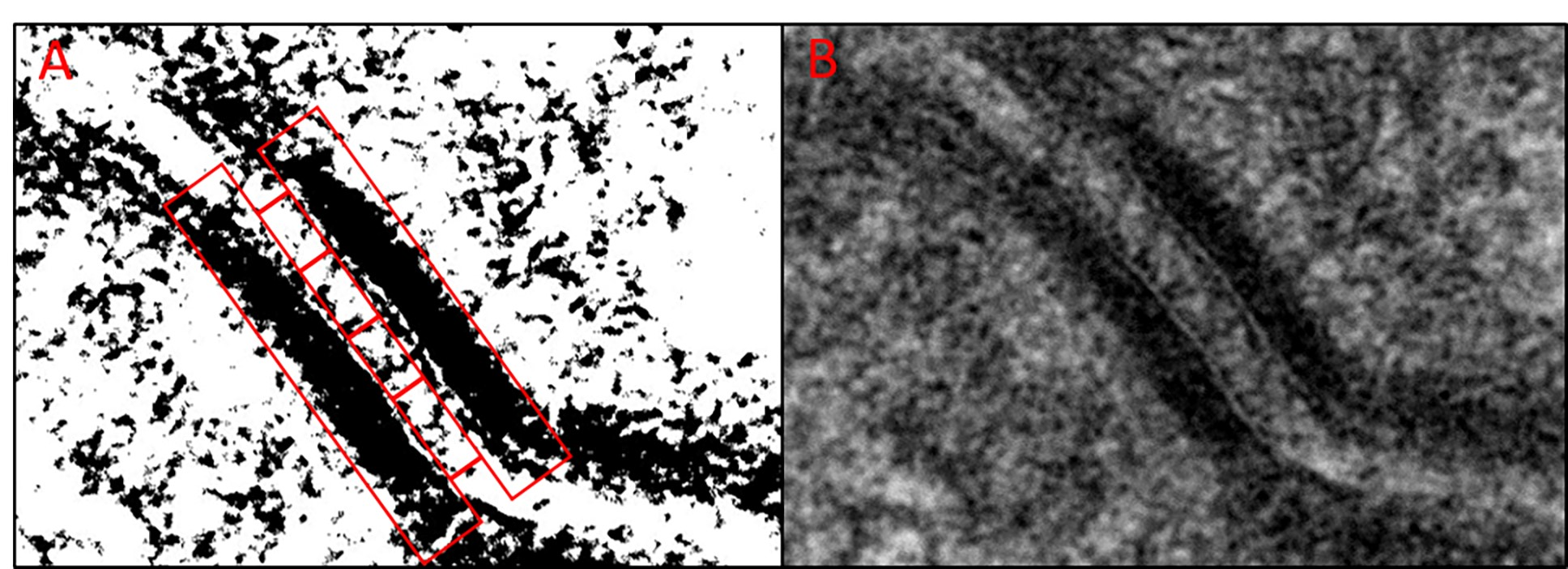


**Supplementary Figure 14: Description of the extracellular space distance measurement in desmosomes.**

The extracellular space distance was measured three times, in five different points, along each desmosome considered in the analysis, at higher magnification. The A panel represents an example of how the distance between two intracellular plaques was measured, using a high contrast black and white image. The B panel shows the original image utilized.

###
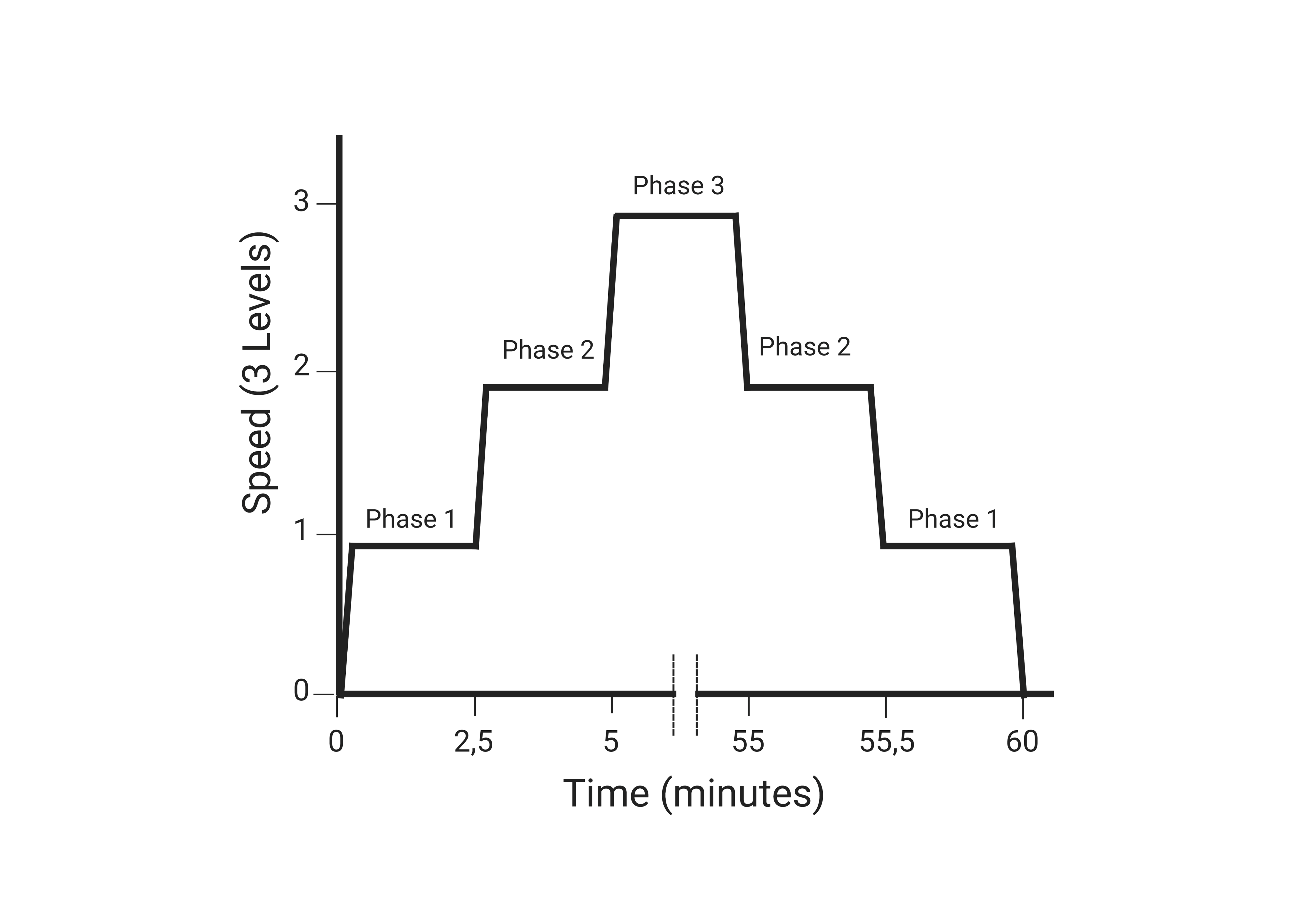


### Supplementary Figure 15: Scheme of the adult training protocol.

### The power of the electric pump in the swim tunnel was divided into three different levels (1/2/3 speed) where 1 corresponds to 11 cm/s, 2 to 12 cm/s and 3 to 13 cm/s output. The training protocol is 1-hour long, including 5 minutes at power level 1 and 2 (acclimation), 50 minutes at level 3 (real intense workout), and 5 minutes at level 2 and 1, returning to rest. The training was repeated 5 days per week for 3 months. (Figure created by Biorender.com)
