# Supplementary material for: A novel DSP zebrafish model reveals training- and drug-induced modulation of arrhythmogenic cardiomyopathy phenotypes
Source: Cell Death Discov. 2023 Dec 6;9:441. doi: 10.1038/s41420-023-01741-2 (PMC10700616; doi:10.1038/s41420-023-01741-2)

**A novel DSP zebrafish model reveals training- and drug-induced modulation of arrhythmogenic cardiomyopathy phenotypes**

**Supplementary File**

**Western blot**

Primary antibody: **Anti Desmoplakin 1/2 (mouse, 651155, Progen, Heidelberg, Germany)** diluted 1:200 in 5% non-fat dry milk TBST.

Secondary antibody: **Anti-mouse IgG (goat) (H+L)-HRP Conjugate (Bio-Rad, Hercules, CA, USA)** diluted 1:2000 in 5% non-fat dry milk TBST.


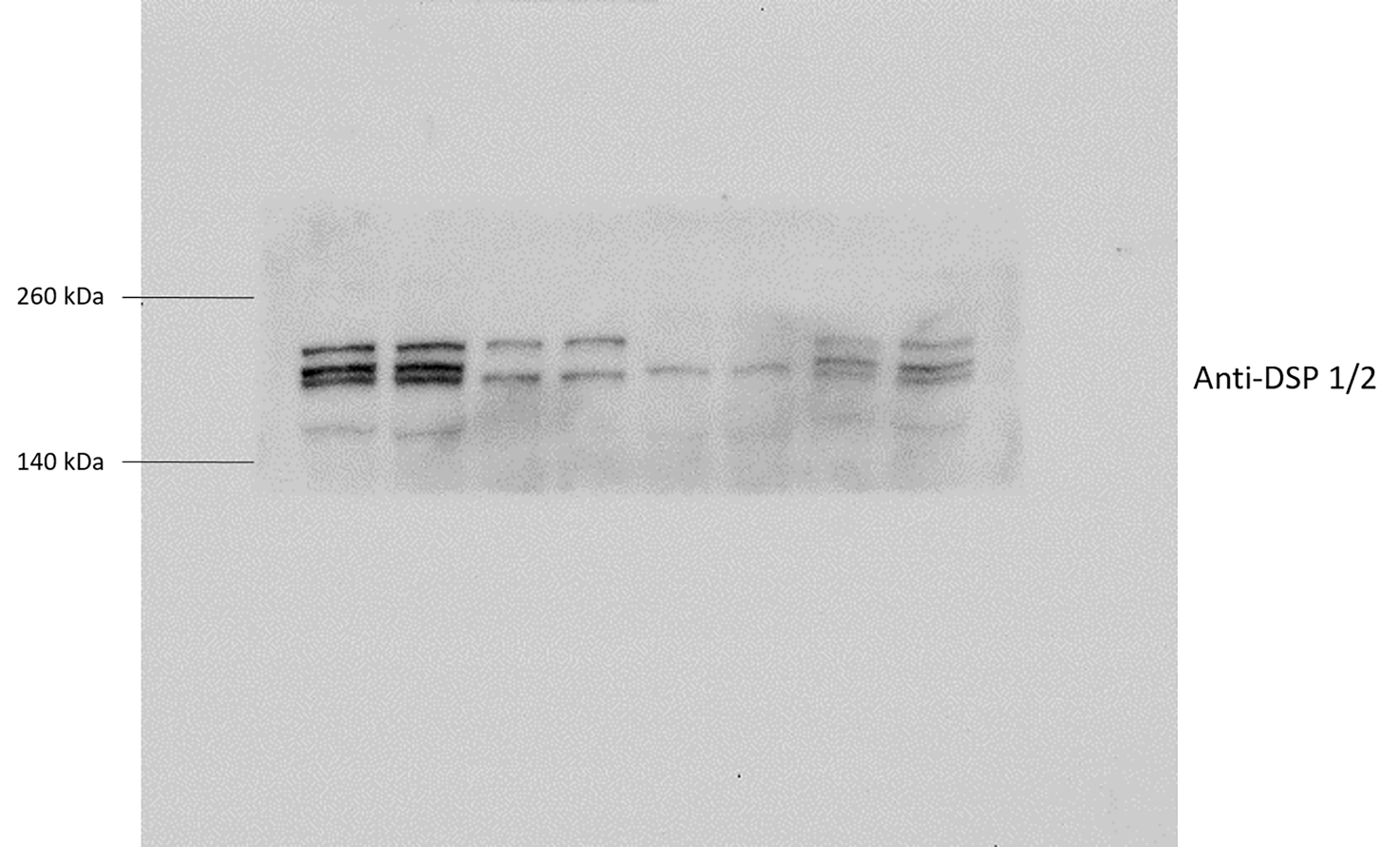


Primary antibody: **Anti α-Tubulin (mouse, A11126, Invitrogen)** diluted 1:1000 in 5% non-fat dry milk TBST.

Secondary antibody: **Anti-mouse IgG (goat) (H+L)-HRP Conjugate (Bio-Rad, Hercules, CA, USA)** diluted 1:2000 in 5% non-fat dry milk TBST.


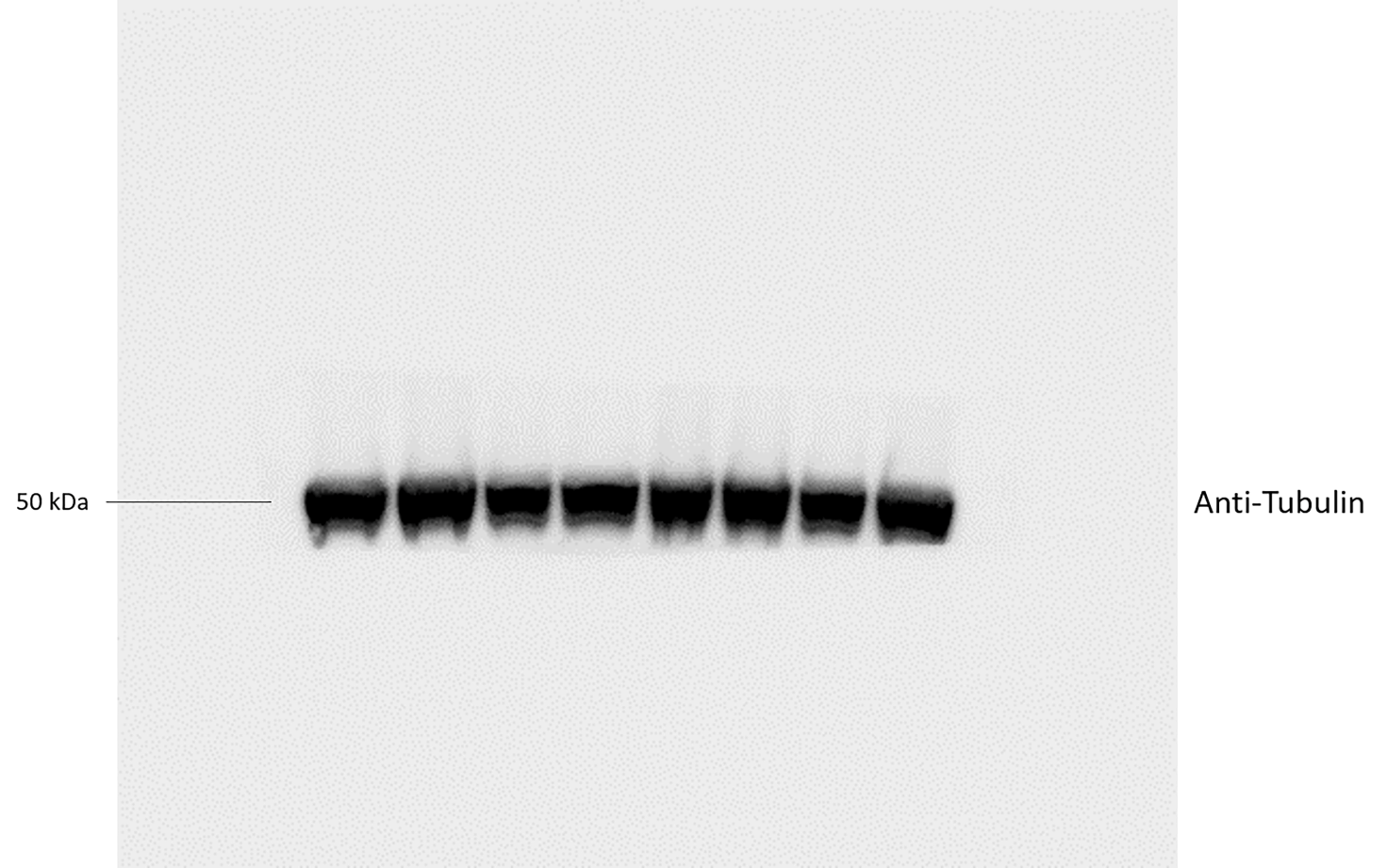

Supplement: Supplementary file 6 — Original Data File [file 41420_2023_1741_MOESM6_ESM.docx]
